# Forecasting extreme temperature events by Hankel-augmented contrastive learning

**DOI:** 10.1093/nsr/nwag456

**Published:** 2026-07-29

**Authors:** Chengyang Qin, Yueyang Ding, Peng Tao, Luonan Chen

**Affiliations:** Key Laboratory of Systems Biology, Hangzhou Institute for Advanced Study University of Chinese Academy of Sciences, Chinese Academy of Sciences, Hangzhou 310024, China; Key Laboratory of Systems Biology, Hangzhou Institute for Advanced Study University of Chinese Academy of Sciences, Chinese Academy of Sciences, Hangzhou 310024, China; Key Laboratory of Systems Biology, Hangzhou Institute for Advanced Study University of Chinese Academy of Sciences, Chinese Academy of Sciences, Hangzhou 310024, China; Key Laboratory of Systems Biology, Hangzhou Institute for Advanced Study University of Chinese Academy of Sciences, Chinese Academy of Sciences, Hangzhou 310024, China; School of Mathematical Sciences and School of AI, Shanghai Jiao Tong University, Shanghai 200240, China

**Keywords:** extreme temperature forecasting, time-series prediction, spatiotemporal representation, augmented learning, contrastive learning

## Abstract

Accurate forecasting of extreme temperature events is critically important for climate adaptation and disaster preparedness, yet it remains a fundamental challenge due to the abrupt and non-stationary nature of such phenomena. Current time-series models often fail to capture rapid transitions and anomalous patterns that deviate significantly from historical behaviors. Here, we propose Hankelformer, a novel deep learning architecture that integrates structured Hankel-based augmentation with contrastive learning to significantly improve the forecasting of extreme weather events. The model employs a structured augmentation module that constructs Hankel matrices to capture local spatiotemporal dynamics without disrupting temporal coherence, generating delay-embedding-inspired views of the original input data in terms of states. These views are processed together with the original input in a dual-stream Transformer encoder, followed by a contrastive learning between them that forms spatiotemporal representations, thus significantly enhancing feature invariance and robustness against distribution shifts. We evaluated Hankelformer on the curated extreme weather datasets: the TexasFreeze, the Pacific Northwest Heatwave and the Antarctic Heat, in addition to six standard benchmarks in energy and transportation. Hankelformer consistently achieves state-of-the-art performance, demonstrating remarkable accuracy in predicting extreme temperature collapses and spikes, with up to 34% improvement in mean squared error over leading baselines. The framework offers a promising tool for reliable extreme temperature forecasting and underscores the value of constructing topologically equivalent sequences to the original sequence for spatiotemporal representation learning in handling real-world non-stationary time series.

## Teaser

By leveraging structured Hankel augmentation and contrastive learning in a dual-stream Transformer, Hankelformer captures local spatiotemporal dynamics for reliable extreme temperature prediction.

## INTRODUCTION

Multivariate time-series (MTS) forecasting underpins critical decisions across domains from energy infrastructure management to climate adaptation, yet failures in prediction can have severe consequences. Accurate forecasting is essential for energy systems requiring precise demand prediction for grid stability and resource allocation [1–3], transportation networks depending on traffic flow forecasting for efficient routing [4,5] and climate monitoring systems relying on robust predictions for disaster preparedness [6–8]. Applications extend to financial risk management, healthcare monitoring and industrial process control [9,10].

Recent extreme events underscore the critical importance of robust forecasting capabilities. The 2021 Texas winter storm exemplifies how inadequate prediction can lead to cascading system failures [11–13]. Economic analyses reveal that electricity demand mispredictions cost the global energy sector billions annually [14,15], while traffic flow prediction errors contribute significantly to congestion-related losses [16,17]. As climate change intensifies extreme event frequency [18,19], developing robust MTS forecasting models capable of handling competitive conditions has become imperative.

The evolution of MTS forecasting has progressed through several paradigmatic shifts. Classical statistical methods such as autoregressive integrated moving average (ARIMA) and vector autoregression (VAR) models [20–22] provided interpretable results but struggled with complex non-linear patterns. Machine learning approaches including Support Vector Machines and Random Forests [23–25] improved non-linear modeling but remained limited in capturing long-range dependencies. The advancement of deep learning introduced recurrent neural networks (RNNs), and long short-term memory (LSTM) networks [26–28], convolutional neural networks (CNNs) for local pattern capture [29,30], and attention mechanisms enabling direct long-range dependency modeling [31,32].

Recent Transformer architectures [32] have shown exceptional promise, spawning innovations including Informer [33], Autoformer [34], TimesNet [35], PatchTST [36], iTransformer [37], FEDformer [38], ETSformer [39], Pyraformer [40], LogTrans [41], Reformer [42] and FiLM [43]. Despite these advances, current approaches face critical limitations: insufficient local pattern extraction during extreme events, limited robustness to distribution shifts [44–46] and suboptimal augmentation strategies that destroy temporal coherence [47,48].

Self-supervised learning has emerged as an important approach with theoretical foundations in information theory [49,50]. Contrastive learning maximizes agreement between augmented views while minimizing agreement with other samples [51–53], significantly advancing computer vision (SimCLR, MoCo) and NLP (BERT variants) [53–55]. Applications to time-series analysis show promise [56,57], with works like TS2Vec [58], CoST [59], TF-C [60], SimMTM [61] and TNC [62] demonstrating improved representations. However, these typically employ generic augmentation strategies potentially destroying temporal coherence and treat self-supervision as separate pretraining, creating representation gaps.

Here, we introduce Hankelformer, addressing these challenges through mathematically principled structured augmentation and end-to-end contrastive learning. Drawing inspiration from delay embedding theorem and Hankel matrix decomposition for spatiotemporal patterns [63–67], we construct topologically equivalent sequences/views to the original sequence/input (see Note S1 for the proof), thus contrastive learning can be efficiently applied between them to form spatiotemporal representations for accurate prediction. Although delay embedding/Hankel matrices and contrastive learning are both well-established techniques, to our knowledge, utilizing Hankel matrices for topologically equivalent augmented representation of time series in conjunction with contrastive learning remains unexplored, constituting one of the core innovations of Hankelformer (see Note S2 for a detailed comparison of Hankelformer with existing contrastive learning methods and delayed embedding methods). Specifically, our approach makes two key innovations: (i) a Hankel-Augmentation (Hankel-AUG) module constructing spatiotemporal patterns/sequences associated with the original sequence through structured sliding windows while maintaining temporal coherence; (ii) a dual-stream contrastive learning framework processing original and augmented sequences with shared-weight Transformer encoders, mitigating potential pretraining-finetuning mismatch. In our context, this mismatch refers to the suboptimality that can arise when representations are first optimized for a generic self-supervised objective and may require substantial adaptation during fine-tuning for the specific downstream forecasting task. The constructed dynamics/sequences for contrastive learning may be topologically equivalent to the original input by the delay embedding theorem or spatiotemporal information transformation [65–69].

The overall Hankelformer architecture is illustrated in Fig. 1. We demonstrate Hankelformer’s effectiveness across nine benchmark datasets, including three custom datasets capturing extreme temperature events: the 2021 North American cold wave (TexasFreeze), the Pacific Northwest heat dome (Heatwave) and the Antarctic Heat. Hankelformer achieves state-of-the-art performance in all scenarios, with strong robustness in extreme event forecasting.

**Figure 1.**
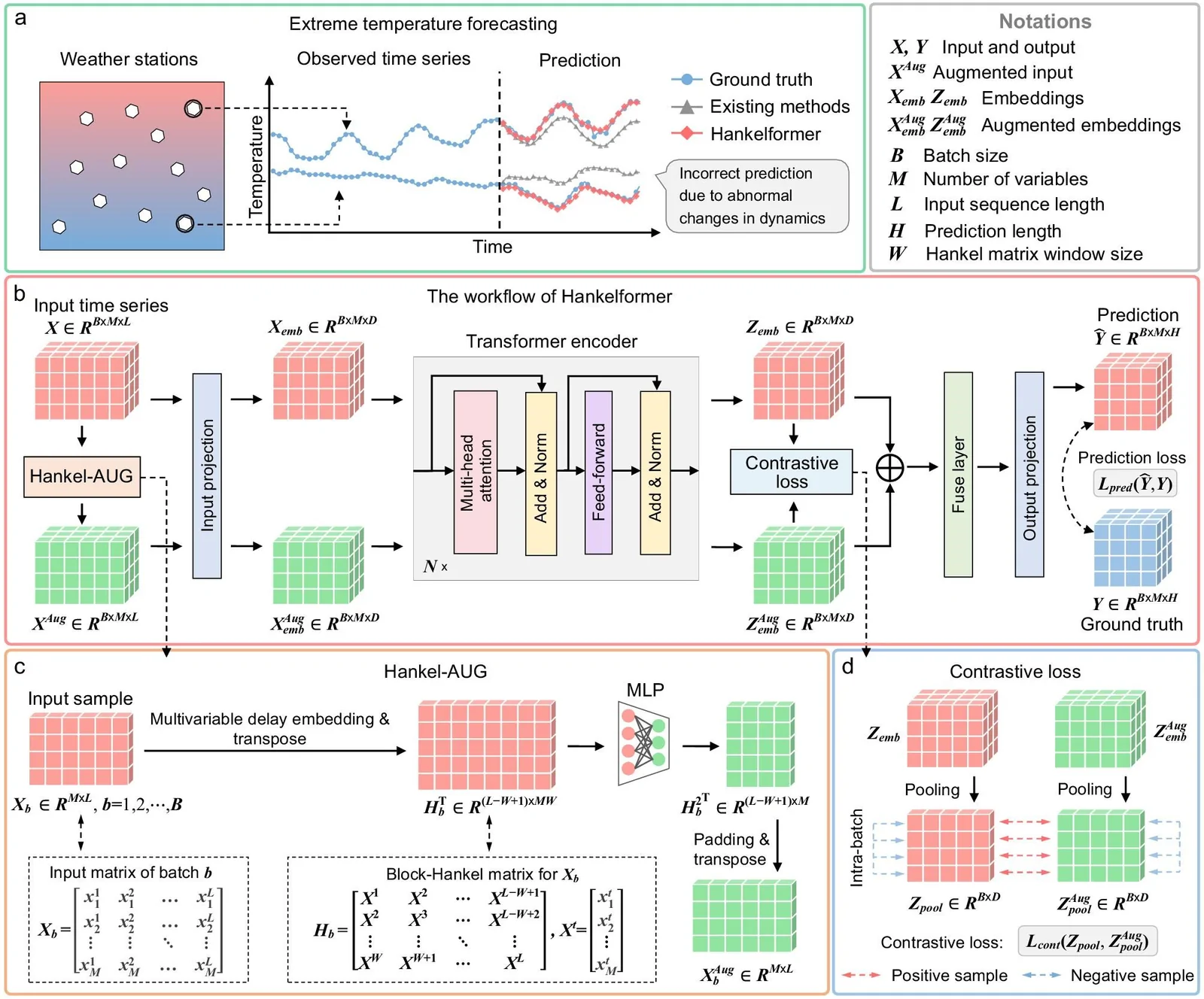
The Hankelformer architecture. (a) Predicting extreme temperature events remains challenging due to abnormal dynamic changes. (b) The overall dual-stream architecture of Hankelformer. The original input sequence ${\boldsymbol{X}}$ is processed in two parallel streams. The top stream processes ${\boldsymbol{X}}$ directly. The bottom stream first passes ${\boldsymbol{X}}$ through the Hankel-AUG module to generate a structurally augmented sequence or view ${{\boldsymbol{X}}}^{{\mathrm{Aug}}}$. Both sequences are embedded and fed into a shared-weight Transformer encoder (*N* layers). The output representations ${{\boldsymbol{Z}}}_{{\mathrm{emb}}}$ and ${\boldsymbol{Z}}_{{\mathrm{emb}}}^{{\mathrm{Aug}}}$ are used to compute a contrastive loss, promoting robust feature learning. The representations are then fused and passed to an output projection layer to generate the final forecast ${\boldsymbol{\hat{Y}}}$. (c) Detailed workflow of the Hankel-AUG module. A block-Hankel matrix is constructed from its original time series/sequence of a specified window size *W*, and its dynamics is topologically equivalent to the original input. The matrix is transformed by a multilayer perceptron (MLP), padded and transposed again to form the Hankel-augmented sequence ${{\boldsymbol{X}}}^{{\mathrm{Aug}}}$. (d) The intra-batch contrastive learning mechanism between the original and augmented sequences. After pooling, the representation of an augmented sample (e.g. green square) is considered a positive sample to its original counterpart (red square) and a negative sample to all other original samples in the batch.

## RESULTS

### The Hankelformer architecture

Given an MTS ${\boldsymbol{X}}\in{\mathbb{R}}^{B \times M \times L}$ with batch size *B, M* variables and *L* historical time steps, the objective is to predict future *H* time steps: ${\boldsymbol{\hat{Y}}}\in{\mathbb{R}}^{B \times M \times H}$. Each time step ${{\boldsymbol{X}}}^t = [ {x_1^t,x_{2,}^t\ldots,x_M^t} ]{\mathrm{\ }}( {t = 1,\ldots,L} )$ represents observations of *M* variables at time *t*.

As illustrated in Fig. 1b, Hankelformer employs a dual-stream architecture that processes original and structurally augmented sequences through shared-weight Transformer encoders. The architecture consists of four core components: Hankel-AUG, dual-stream encoding, contrastive learning and feature fusion.

*Hankel-AUG module.* The Hankel-AUG module (Fig. 1c) generates structurally augmented views/sequences by extracting local spatiotemporal patterns through Hankel matrix decomposition. For each sample ${{\boldsymbol{X}}}_b\in{\mathbb{R}}^{M \times L}$ within the batch *B*, where $b = 1,\ 2,\ldots,B$, it is transformed into a block-Hankel matrix by multivariable delayed embedding,


(1) \begin{eqnarray*}
{{\boldsymbol{H}}}_b = \left[ {\begin{array}{@{}c@{\quad}c@{\quad}c@{\quad}c@{}} {{{\boldsymbol{X}}}^1}&{{{\boldsymbol{X}}}^2}& \cdots &{{{\boldsymbol{X}}}^{L - W + 1}}\\ {{{\boldsymbol{X}}}^2}&{{{\boldsymbol{X}}}^3}& \cdots &{{{{\mathbf{X}}}}^{L - W + 2}}\\ \vdots & \vdots & \ddots & \vdots \\ {{{\boldsymbol{X}}}^W}&{{{\boldsymbol{X}}}^{W + 1}}& \cdots &{{{\boldsymbol{X}}}^L} \end{array}} \right],
\end{eqnarray*}

where *W* is the window size. This yields a tensor ${{\boldsymbol{H}}}_b\in{\mathbb{R}}^{MW \times ( {L - W + 1} )}$ and it is transposed to ${\mathbb{R}}^{( {L - W + 1} ) \times MW}$, processed through an MLP, padded and transposed to generate ${\boldsymbol{X}}_{\boldsymbol{b}}^{{{\mathbf{Aug}}}}\in{\mathbb{R}}^{M \times L}$, maintaining dimensional compatibility while capturing complementary local patterns. Note that the Hankel matrix is one delay embedding form of the original time series based on delay embedding theorem (see Note S1 for the proof), and thus its dynamics is topologically equivalent to the original one and also considered as a temporal/dynamical representation of the original time series [63–69].

*Dual-stream encoding*. The original input ${\boldsymbol{X}}$ undergoes two parallel pathways. The upper stream processes multivariate ${\boldsymbol{X}}$ directly through input projection to obtain ${{\boldsymbol{X}}}_{{\mathrm{emb}}}\in{\mathbb{R}}^{B \times M \times D}$. The lower stream first applies the Hankel-AUG module in the delay embedding form to generate ${{\boldsymbol{X}}}^{{\mathrm{Aug}}}$, followed by input projection to obtain ${\boldsymbol{X}}_{{\mathrm{emb}}}^{{\mathrm{Aug}}}\in{\mathbb{R}}^{B \times M \times D}$, where *D* is the model dimension. Both embedded sequences are processed through a shared-weight multilayer Transformer encoder, yielding representations ${{\boldsymbol{Z}}}_{{\mathrm{emb}}}\in{\mathbb{R}}^{B \times M \times D}$ and ${\boldsymbol{Z}}_{{\mathrm{emb}}}^{{\mathrm{Aug}}}\in{\mathbb{R}}^{B \times M \times D}$. Note that the two representations originate from two topologically equivalent inputs (${\boldsymbol{X}}$ and its corresponding Hankel matrix), and thus contrastive learning can be efficiently applied to construct the spatiotemporal representation of the original MTS.

*Contrastive learning*. After average pooling of ${{\boldsymbol{Z}}}_{{\mathrm{emb}}}\ and\ {\boldsymbol{Z}}_{{\mathrm{emb}}}^{{\mathrm{Aug}}}$, we obtain ${{\boldsymbol{Z}}}_{{\mathrm{pool}}}{\mathrm{\ }}\in{\mathbb{R}}^{B \times D}\ {\mathrm{and}}\ {\boldsymbol{Z}}_{{\mathrm{pool\ }}}^{{\mathrm{Aug\ }}}\in{\mathbb{R}}^{B \times D}$. The contrastive learning objective maximizes agreement between original and augmented representations while minimizing agreement with other samples in the batch. Given the batch size *B* with samples indexed by $i,j\in\{ {1,\ 2,\ldots,B} \}$, a sample’s representation with its augmentation (${z}_i\in{{\boldsymbol{Z}}}_{{\mathrm{pool\ }}},z_i^{{\mathrm{Aug\ }}}\in{\boldsymbol{Z}}_{{\mathrm{pool\ }}}^{{\mathrm{Aug\ }}}$) are defined as a positive pair. Conversely, negative pairs consist of a given ${z}_i$ and all other augmented representations ${\{ {z_j^{{\mathrm{Aug}}}} \}}_{j\ne i}$ within the same batch. Both original and augmented representations are first L2-normalized as


(2) \begin{eqnarray*}
{z}_i = \frac{{{z}_i}}{{\|{z}_i{\|}_2}},\quad z_i^{{\mathrm{Aug}}} = \frac{{z_i^{{\mathrm{Aug}}}}}{{\|z_i^{{\mathrm{Aug}}}{\|}_2}},
\end{eqnarray*}

where $\| \cdot {\|}_2$ denotes the Euclidean norm. Finally, inspired by the work of Gao *et al.* [55], the contrastive loss ${L}_{{\mathrm{cont}}}$ is defined as


(3) \begin{eqnarray*}
{L}_{{\mathrm{cont}}}{\mathrm{\ }} = - \frac{1}{B}\mathop \sum \limits_{i = 1}^B \log \left( {\frac{{\exp \left( {\frac{{{\mathrm{sim}}\left( {{z}_i,z_i^{{\mathrm{Aug}}}{\mathrm{\ }}} \right)}}{\tau }} \right)}}{{\mathop \sum \nolimits_{j = 1}^B \exp \left( {\frac{{{\mathrm{sim}}\left( {{z}_i,z_j^{{\mathrm{Aug}}}{\mathrm{\ }}} \right)}}{\tau }} \right)}}} \right),
\end{eqnarray*}

where ${\mathrm{sim}}( { \cdot , \cdot } )$ denotes cosine similarity and *τ * is the temperature parameter.

*Feature fusion*. The fused representation combines information from both streams as the spatiotemporal representation:


(4) \begin{eqnarray*}
{\boldsymbol{Z}}_{{\mathrm{emb}}}^{{\mathrm{fuse}}} = \textit{LinearFusion}\ \left( {\textit{Concat}\ \left( {{\boldsymbol{Z}}_{{\mathrm{emb}}}^{{\mathrm{Aug}}},{{\boldsymbol{Z}}}_{{\mathrm{emb}}}{\mathrm{\ }}} \right)} \right).
\end{eqnarray*}

Final predictions are obtained through output projection:


(5) \begin{eqnarray*}
{\boldsymbol{\hat{Y}}} = \textit{OutputProjection}\ \left( {{\boldsymbol{Z}}_{{\mathrm{emb}}}^{{\mathrm{fuse}}}} \right).
\end{eqnarray*}

The total loss function integrates forecasting and contrastive objectives:


(6) \begin{eqnarray*}
{L}_{{\mathrm{total}}}{\mathrm{\ }} = {L}_{{\mathrm{pred}}} ( {{{\bf \hat{Y}}},{{\mathbf{Y}}}}) + \lambda \cdot {L}_{{\mathrm{cont}}},
\end{eqnarray*}

where ${L}_{{\mathrm{pred}}}$ is the mean squared error (MSE) and *λ * is a weight parameter of the contrastive objective.

### Validation of Hankelformer on a chaotic 90D-Lorenz system

To validate the effectiveness of Hankelformer, we first evaluated its predictive performance on a 90-dimensional (90D) chaotic Lorenz system, whose ground-truth solution is known. Using 96 observed time steps, we forecasted the next 96 steps and compared Hankelformer against several representative baselines: WPMixer, iTransformer, TimeMixer, TiDE, PatchTST, TimesNet, CoST, SimMTM and Naïve (consistently utilizes the last value as the prediction), covering Transformer-based architectures, decomposition-based models and MLP/Mixer-style methods. Figure 2a and b compares the predictions for two example Lorenz dimensions and the predictions of Hankelformer align closest to the true dynamics. Quantitatively, Hankelformer achieves an MSE of 0.348 and an mean absolute error (MAE) of 0.380, outperforming the second-best method, TimeMixer, which records 0.405 and 0.424, respectively (Table 1).

**Figure 2.**
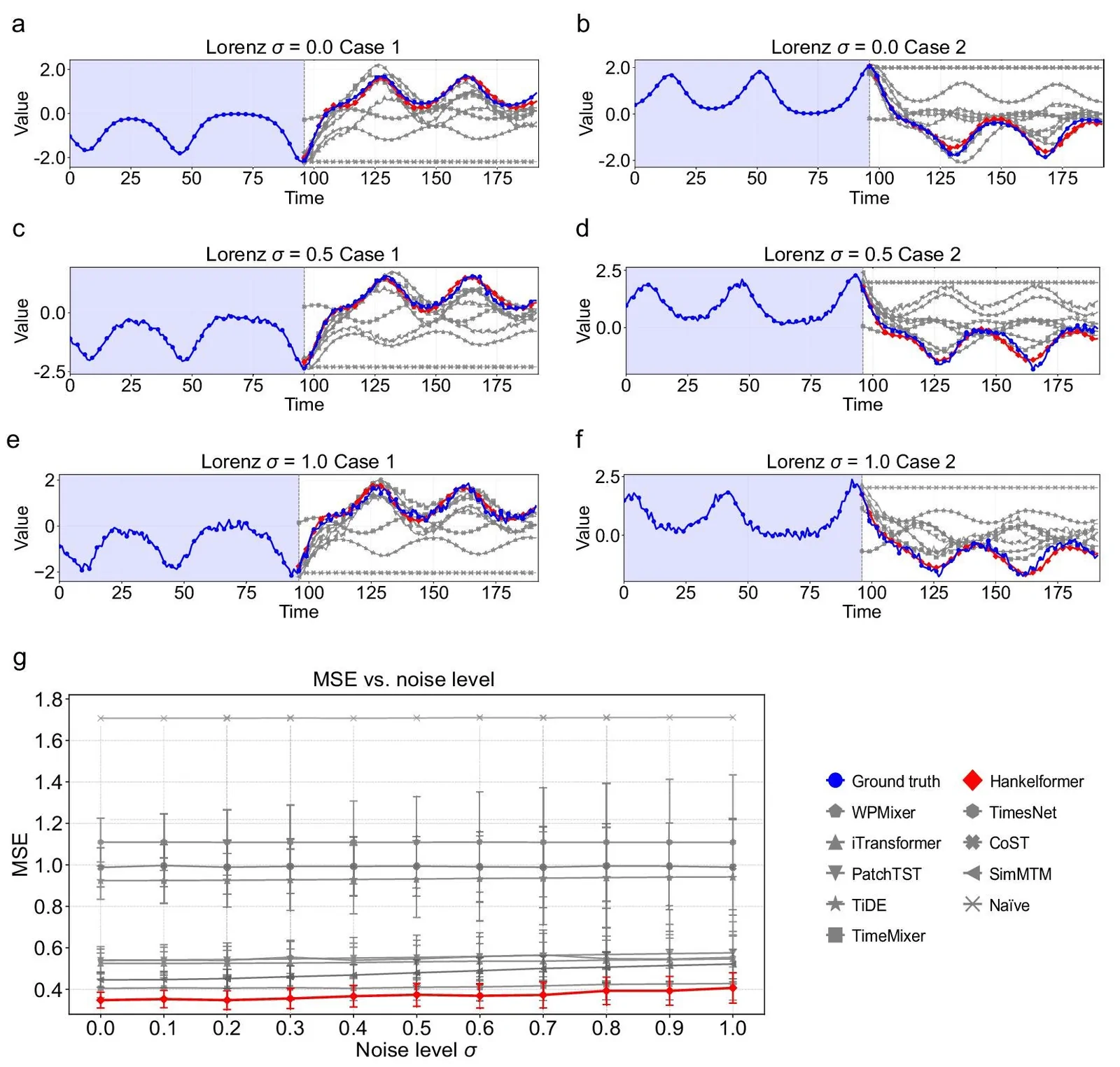
Hankelformer demonstrates superior robustness to noise on the chaotic Lorenz system. (a–f) Qualitative prediction results of two example dimensions for noise levels *σ = 0*, *σ = 0.5* and *σ = 1*, respectively. As noise increases, Hankelformer’s predictions remain stable and accurate, while competitors' predictions diverge significantly. (g) Quantitative plot showing MSE as a function of noise level. The error bar represents the range of prediction error across 90 variables. Hankelformer’s error is consistently the lowest and exhibits the slowest rate of increase, highlighting its superior noise robustness.

**Table 1.** Evaluation of Hankelformer on the 90D-Lorenz dataset. Forecasting performance (MSE/MAE) under increasing levels of additive Gaussian noise (*σ * from 0.0 to 1.0). All models predict 96 future steps based on 96 past steps. Best results are in bold. ‘1st Cnt’ indicates the number of times a model achieved the best score.

	Hankelformer (Ours)		WPMixer (2025)		iTransformer (2024)		TimeMixer (2024)		TiDE (2023)		PatchTST (2023)		SimMTM (2023)		CoST (2022)		TimesNet (2022)		Naïve	
σ	MSE	MAE	MSE	MAE	MSE	MAE	MSE	MAE	MSE	MAE	MSE	MAE	MSE	MAE	MSE	MAE	MSE	MAE	MSE	MAE
0	**0.348**	**0.380**	0.539	0.515	0.525	0.506	0.405	0.424	0.924	0.720	0.541	0.520	0.446	0.449	0.988	0.799	1.110	0.855	1.707	1.011
0.1	**0.353**	**0.384**	0.541	0.516	0.525	0.507	0.407	0.425	0.925	0.721	0.542	0.520	0.447	0.450	0.997	0.801	1.110	0.854	1.707	1.011
0.2	**0.348**	**0.381**	0.540	0.516	0.526	0.507	0.406	0.424	0.926	0.722	0.544	0.522	0.452	0.455	0.989	0.799	1.109	0.854	1.707	1.011
0.3	**0.356**	**0.386**	0.555	0.523	0.527	0.509	0.408	0.426	0.928	0.724	0.547	0.525	0.461	0.462	0.993	0.799	1.109	0.854	1.708	1.011
0.4	**0.367**	**0.396**	0.540	0.516	0.529	0.510	0.405	0.426	0.930	0.726	0.551	0.527	0.469	0.469	0.993	0.802	1.109	0.854	1.707	1.011
0.5	**0.374**	**0.401**	0.547	0.524	0.533	0.512	0.410	0.429	0.932	0.728	0.554	0.530	0.480	0.478	0.994	0.801	1.109	0.854	1.708	1.012
0.6	**0.369**	**0.398**	0.558	0.533	0.536	0.515	0.412	0.432	0.935	0.730	0.559	0.534	0.490	0.486	0.991	0.800	1.110	0.854	1.710	1.013
0.7	**0.373**	**0.402**	0.566	0.538	0.536	0.516	0.417	0.437	0.936	0.731	0.564	0.537	0.501	0.494	0.989	0.799	1.109	0.854	1.709	1.013
0.8	**0.393**	**0.417**	0.548	0.521	0.541	0.520	0.424	0.442	0.939	0.733	0.568	0.540	0.507	0.500	0.995	0.800	1.109	0.854	1.710	1.013
0.9	**0.393**	**0.418**	0.546	0.522	0.543	0.521	0.426	0.444	0.941	0.734	0.572	0.543	0.515	0.507	0.994	0.802	1.109	0.854	1.711	1.014
1.0	**0.407**	**0.428**	0.554	0.529	0.547	0.525	0.428	0.447	0.942	0.736	0.576	0.547	0.522	0.512	0.989	0.799	1.109	0.854	1.711	1.015
1st Cnt	**11**	**11**	0	0	0	0	0	0	0	0	0	0	0	0	0	0	0	0	0	0

To further assess the robustness of Hankelformer, we injected Gaussian noise of controlled intensity during Lorenz simulation (see ‘Methods’ section). Figure 2c–f compares the predictions under noise levels of 0.5 and 1.0, while Fig. 2g summarizes the MSE and corresponding standard deviations under different noise conditions. Regardless of noise intensity, Hankelformer consistently incurs significantly lower error than the those competing methods, corroborating its noise robustness (Table 1).

In addition, to evaluate the out-of-distribution (OOD) generalization performance of Hankelformer, we added a cross-system experiment. Specifically, all models were trained exclusively on trajectories generated from a 90D coupled Lorenz system and then evaluated directly, without retraining, on trajectories generated from a 90D coupled Rössler system. No Rössler samples were used during training or hyperparameter optimization. The Lorenz and Rössler systems exhibit substantially different chaotic dynamics and attractor structures, making this zero-shot cross-system transfer setting a controlled OOD benchmark. The results are summarized in Table S1 and Fig. S1. Although all methods exhibit significant prediction errors when evaluated on the Rössler system without retraining, Hankelformer still demonstrates relatively superior performance. These results provide empirical evidence that the proposed Hankel-augmented contrastive framework learns representations that are more transferable under certain non-linear distribution shifts compared with standard forecasting architectures.

### Evaluation of Hankelformer on six public datasets

We next conducted a systematic evaluation of Hankelformer on six public datasets from the energy and transportation domains: Electricity (ECL), Traffic and PEMS03/04/07/08. For ECL and Traffic, we employed an input window of 96 time steps to forecast horizons of 96, 192, 336 and 720 steps; for the PEMS datasets, the model kept an input window of 96 time steps and predicted 12, 24, 48 and 96 steps ahead. Table 2 summarizes the MSE and MAE values for Hankelformer and all baseline models. Hankelformer consistently achieves the lowest errors across all datasets and forecast horizons.

**Table 2.** Main forecasting results on six public benchmark datasets. Forecasting performance (MSE/MAE) for multistep forecasting across various horizons. Predictions are based on 96 historical time steps. Best results for each metric and horizon are highlighted in bold. The ‘Avg’ row reports the average score across all horizons. ‘1st Cnt’ indicates the number of times a model achieved the best score.

		Hankelformer (Ours)		WPMixer (2025)		iTransformer (2024)		TimeMixer (2024)		TiDE (2023)		PatchTST (2023)		SimMTM (2023)		CoST (2022)		TimesNet (2022)		Naïve	
Dataset	Time step	MSE	MAE	MSE	MAE	MSE	MAE	MSE	MAE	MSE	MAE	MSE	MAE	MSE	MAE	MSE	MAE	MSE	MAE	MSE	MAE
ECL	96	**0.141**	**0.237**	0.150	0.241	0.148	0.240	0.153	0.247	0.237	0.329	0.181	0.270	0.164	0.255	0.275	0.372	0.168	0.272	1.588	0.946
	192	**0.157**	**0.252**	0.162	**0.252**	0.162	0.253	0.166	0.256	0.236	0.330	0.188	0.274	0.178	0.268	0.284	0.382	0.184	0.289	1.595	0.950
	336	**0.169**	**0.265**	0.179	0.270	0.178	0.269	0.185	0.277	0.249	0.344	0.204	0.293	0.190	0.280	0.297	0.393	0.198	0.300	1.617	0.961
	720	**0.199**	**0.296**	0.217	0.304	0.225	0.317	0.225	0.310	0.284	0.373	0.246	0.324	0.235	0.318	0.303	0.393	0.220	0.320	1.647	0.975
	Avg	**0.167**	**0.263**	0.177	0.267	0.178	0.270	0.182	0.273	0.252	0.344	0.205	0.290	0.192	0.280	0.290	0.385	0.193	0.295	1.612	0.958
Traffic	96	**0.395**	**0.266**	0.465	0.286	**0.395**	0.268	0.462	0.285	0.805	0.493	0.649	0.389	0.550	0.353	0.735	0.445	0.593	0.321	2.723	1.079
	192	**0.413**	**0.273**	0.475	0.290	0.417	0.276	0.473	0.296	0.756	0.474	0.601	0.366	0.563	0.365	0.719	0.425	0.617	0.336	2.756	1.087
	336	**0.423**	**0.278**	0.489	0.296	0.433	0.283	0.498	0.296	0.762	0.477	0.609	0.369	0.587	0.368	0.735	0.438	0.629	0.336	2.789	1.094
	720	**0.463**	**0.300**	0.527	0.318	0.467	0.302	0.506	0.313	0.719	0.449	0.647	0.387	0.602	0.385	0.756	0.445	0.640	0.350	2.810	1.097
	Avg	**0.424**	**0.279**	0.489	0.298	0.428	0.282	0.485	0.298	0.761	0.473	0.627	0.378	0.576	0.368	0.736	0.438	0.620	0.336	2.770	1.089
PEMS03	96	**0.063**	**0.169**	0.071	0.178	0.071	0.174	0.078	0.188	0.178	0.305	0.099	0.216	0.082	0.189	0.116	0.229	0.593	0.192	0.136	0.250
	192	**0.086**	**0.196**	0.108	0.219	0.093	0.201	0.116	0.230	0.257	0.371	0.142	0.259	0.133	0.239	0.135	0.250	0.617	0.223	0.265	0.350
	336	**0.121**	**0.233**	0.202	0.300	0.125	0.236	0.200	0.301	0.379	0.463	0.211	0.319	0.233	0.326	0.253	0.365	0.629	0.260	0.596	0.544
	720	**0.138**	**0.252**	0.293	0.362	0.164	0.275	0.326	0.390	0.490	0.539	0.269	0.370	0.247	0.332	0.254	0.365	0.640	0.317	1.291	0.857
	Avg	**0.102**	**0.213**	0.169	0.265	0.113	0.222	0.180	0.277	0.326	0.420	0.180	0.291	0.174	0.272	0.190	0.302	0.620	0.248	0.572	0.500
PEMS04	96	**0.072**	**0.177**	0.083	0.188	0.078	0.183	0.093	0.204	0.219	0.340	0.105	0.224	0.100	0.205	0.111	0.221	0.085	0.195	0.157	0.269
	192	**0.080**	**0.187**	0.118	0.229	0.095	0.205	0.133	0.247	0.292	0.398	0.153	0.275	0.125	0.241	0.119	0.233	0.118	0.215	0.289	0.372
	336	**0.096**	**0.209**	0.205	0.307	0.120	0.233	0.241	0.343	0.409	0.478	0.229	0.339	0.190	0.293	0.149	0.267	0.155	0.250	0.642	0.572
	720	**0.106**	**0.219**	0.287	0.367	0.150	0.262	0.336	0.407	0.492	0.532	0.291	0.389	0.201	0.299	0.172	0.293	0.228	0.303	1.410	0.904
	Avg	**0.089**	**0.198**	0.173	0.273	0.111	0.221	0.201	0.300	0.353	0.437	0.195	0.307	0.154	0.260	0.138	0.254	0.147	0.241	0.625	0.529
PEMS07	96	**0.058**	**0.152**	0.066	0.165	0.067	0.165	0.072	0.178	0.173	0.304	0.095	0.207	0.072	0.180	0.176	0.253	0.087	0.181	0.132	0.247
	192	**0.072**	**0.172**	0.111	0.219	0.088	0.190	0.107	0.215	0.271	0.383	0.150	0.262	0.111	0.220	0.180	0.261	0.103	0.204	0.273	0.355
	336	**0.093**	**0.197**	0.198	0.292	0.110	0.215	0.178	0.273	0.446	0.495	0.253	0.340	0.191	0.291	0.202	0.288	0.136	0.238	0.645	0.557
	720	**0.112**	**0.214**	0.312	0.368	0.139	0.245	0.278	0.342	0.628	0.577	0.346	0.404	0.198	0.302	0.255	0.339	0.190	0.279	1.382	0.872
	Avg	**0.084**	**0.184**	0.172	0.261	0.101	0.204	0.159	0.252	0.380	0.440	0.211	0.303	0.143	0.248	0.203	0.285	0.129	0.226	0.608	0.508
PEMS08	96	**0.074**	**0.177**	0.086	0.189	0.079	0.182	0.093	0.201	0.227	0.343	0.168	0.232	0.110	0.223	0.255	0.280	0.112	0.212	0.148	0.263
	192	**0.103**	**0.209**	0.136	0.243	0.115	0.219	0.143	0.250	0.318	0.409	0.224	0.281	0.182	0.289	0.271	0.295	0.141	0.238	0.283	0.369
	336	**0.166**	**0.228**	0.245	0.322	0.186	0.235	0.263	0.344	0.497	0.510	0.321	0.354	0.287	0.333	0.340	0.355	0.198	0.283	0.655	0.577
	720	**0.211**	**0.259**	0.390	0.389	0.221	0.267	0.435	0.432	0.721	0.592	0.408	0.417	0.302	0.349	0.409	0.407	0.320	0.351	1.461	0.909
	Avg	**0.139**	**0.218**	0.214	0.286	0.150	0.226	0.234	0.307	0.441	0.464	0.280	0.321	0.220	0.299	0.319	0.334	0.193	0.271	0.637	0.530
1st Cnt		**30**	**30**	0	1	1	0	0	0	0	0	0	0	0	0	0	0	0	0	0	0

On the highly volatile electricity load series (Fig. 3a and b), Hankelformer not only captures the dominant trend but also accurately reproduces the true peaks and troughs, whereas the predictions from baseline models exhibit noticeable deviations. Similarly, on the normalized traffic flow series (Fig. 3c–l), Hankelformer maintains comparable superiority. These results confirm Hankelformer’s ability to model long-range dependencies and complex non-linear dynamics, demonstrating strong generalization and robustness across diverse domains and data characteristics.

**Figure 3.**
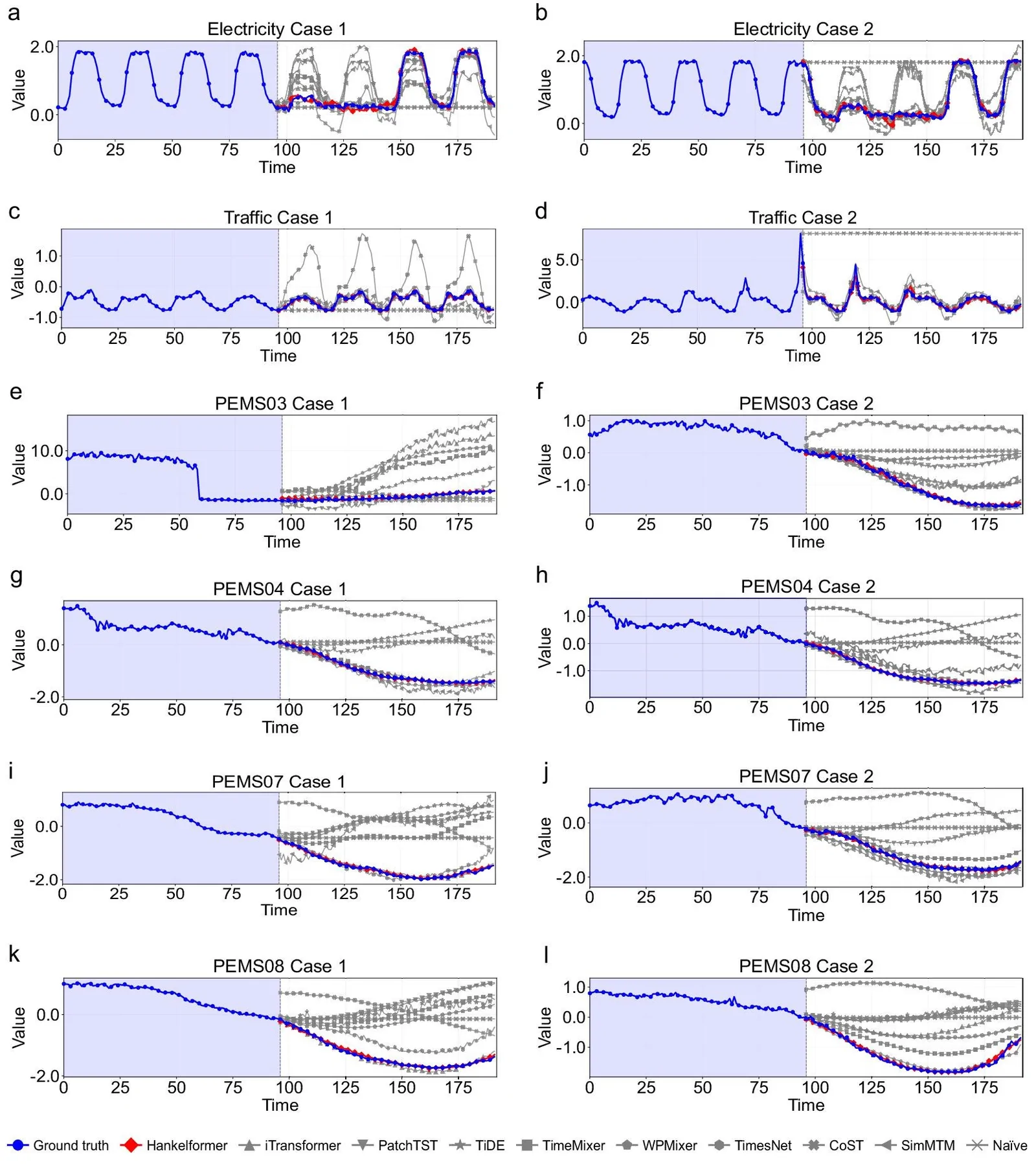
Forecasting results on standard benchmark datasets. The figure displays representative 96-step ahead forecasting results on various datasets. (a and b) Two distinct cases from the ECL dataset. (c and d) Two cases from the Traffic dataset. (e and f) Two cases from the PEMS03 dataset. (g and h) Two cases from the PEMS04 dataset. (i and j) Two cases from the PEMS07 dataset. (k and l) Two cases from the PEMS08 dataset. Across all scenarios, Hankelformer demonstrates a superior ability to align with the ground truth compared to other models.

### Forecasting of extreme temperature events

Accurate forecasting of extreme temperature events remains one of the most critical yet challenging tasks in meteorological science. Conventional time-series models often rely on the assumption of weak stationarity, which limits their ability to capture abrupt onsets and extreme amplitudes characteristic of such phenomena. Consequently, developing forecasting tools that remain reliable under extreme conditions is of significant scientific and societal value. To evaluate Hankelformer’s performance in such scenarios, we constructed three tailored datasets based on recent real-world events: TexasFreeze (the February 2021 North American cold wave), Heatwave (the June 2021 Pacific Northwest heat dome) and Antarctic Heat (the February 2020 Antarctic Peninsula heat event). As summarized in Table 3, Hankelformer achieves the lowest MSE and MAE across all forecast horizons and outperforms most baseline models.

**Table 3.** Forecasting results on extreme temperature datasets. Performance (MSE/MAE) for multistep temperature prediction on the TexasFreeze, Heatwave and Antarctic Heat datasets. Predictions are based on 96 past hours. Best results are in bold. The ‘Avg’ row reports the average score across all horizons. ‘1st Cnt’ indicates the number of times a model achieved the best score.

		Hankelformer (Ours)		WPMixer (2025)		iTransformer (2024)		TimeMixer (2024)		TiDE (2023)		PatchTST (2023)		SimMTM (2023)		CoST (2022)		TimesNet (2022)		Naïve	
Dataset	Time step	MSE	MAE	MSE	MAE	MSE	MAE	MSE	MAE	MSE	MAE	MSE	MAE	MSE	MAE	MSE	MAE	MSE	MAE	MSE	MAE
TexasFreeze	12	**0.105**	**0.212**	0.160	0.269	0.140	0.251	0.153	0.257	0.165	0.280	0.159	0.277	0.109	0.210	0.117	0.225	0.163	0.280	0.287	0.353
	24	**0.179**	**0.296**	0.281	0.380	0.250	0.360	0.265	0.361	0.278	0.384	0.271	0.378	0.185	0.296	0.221	0.254	0.221	0.334	0.379	0.426
	48	**0.347**	**0.431**	0.535	0.545	0.551	0.548	0.518	0.532	0.521	0.542	0.510	0.535	0.358	0.439	0.361	0.442	0.330	0.432	0.541	0.529
	96	**0.628**	0.619	1.086	0.805	1.108	0.807	0.982	0.772	1.035	0.798	1.055	0.804	0.629	**0.617**	0.667	0.653	0.698	0.644	0.930	0.726
	Avg	**0.315**	**0.390**	0.516	0.500	0.512	0.492	0.480	0.481	0.500	0.501	0.499	0.499	0.320	0.391	0.342	0.394	0.353	0.423	0.534	0.509
Heatwave	12	**0.100**	**0.205**	0.150	0.255	0.128	0.236	0.141	0.246	0.158	0.269	0.158	0.269	0.109	0.214	0.103	0.218	0.121	0.238	0.927	0.747
	24	**0.170**	**0.276**	0.221	0.320	0.189	0.292	0.213	0.310	0.224	0.327	0.229	0.331	0.181	0.280	0.192	0.289	0.172	0.285	0.944	0.751
	48	**0.263**	0.358	0.338	0.413	0.305	0.388	0.331	0.398	0.333	0.413	0.340	0.416	0.271	**0.355**	0.303	0.358	0.316	0.386	1.017	0.784
	96	**0.451**	**0.466**	0.472	0.488	0.499	0.506	0.479	0.485	0.494	0.506	0.486	0.500	0.465	0.469	0.472	0.493	0.613	0.555	1.180	0.839
	Avg	**0.246**	**0.326**	0.295	0.369	0.280	0.356	0.291	0.360	0.302	0.379	0.303	0.379	0.257	0.330	0.268	0.340	0.306	0.366	1.017	0.780
Antarctic Heat	12	**0.147**	**0.263**	0.168	0.277	0.156	0.268	0.161	0.271	0.176	0.281	0.168	0.277	0.171	0.284	0.161	0.291	0.175	0.299	0.524	0.491
	24	**0.203**	**0.320**	0.234	0.342	0.212	0.326	0.222	0.332	0.233	0.338	0.227	0.337	0.229	0.341	0.229	0.328	0.221	0.343	0.583	0.537
	48	**0.272**	**0.382**	0.290	0.394	0.275	0.384	0.279	0.387	0.289	0.391	0.284	0.390	0.291	0.397	0.294	0.399	0.288	0.399	0.689	0.597
	96	**0.316**	**0.419**	0.331	0.428	0.349	0.442	0.324	0.423	0.337	0.431	0.327	0.425	0.337	0.432	0.348	0.426	0.325	0.427	0.783	0.645
	Avg	**0.235**	**0.346**	0.256	0.360	0.248	0.355	0.247	0.353	0.259	0.360	0.252	0.357	0.257	0.364	0.258	0.361	0.252	0.367	0.645	0.568
1st Cnt		**15**	**13**	0	0	0	0	0	0	0	0	0	0	0	2	0	0	0	0	0	0

Specifically, during the TexasFreeze cold surge (Fig. 4a and d), Hankelformer accurately reproduces the precipitous temperature drop and subsequent recovery, whereas the baseline models systematically underestimate both the rate and magnitude of the decline. Similarly, during the Heatwave and Antarctic Heat event (Fig. 4b, c, e and f), Hankelformer closely tracks the anomalous temperature spike with high fidelity, while competing models exhibit noticeable deviations. These results demonstrate that Hankelformer generalizes effectively across radically different and highly non-linear extreme weather patterns. Notably, the performance advantage of Hankelformer becomes more pronounced at longer forecasting horizons (Fig. 4g–i).

**Figure 4.**
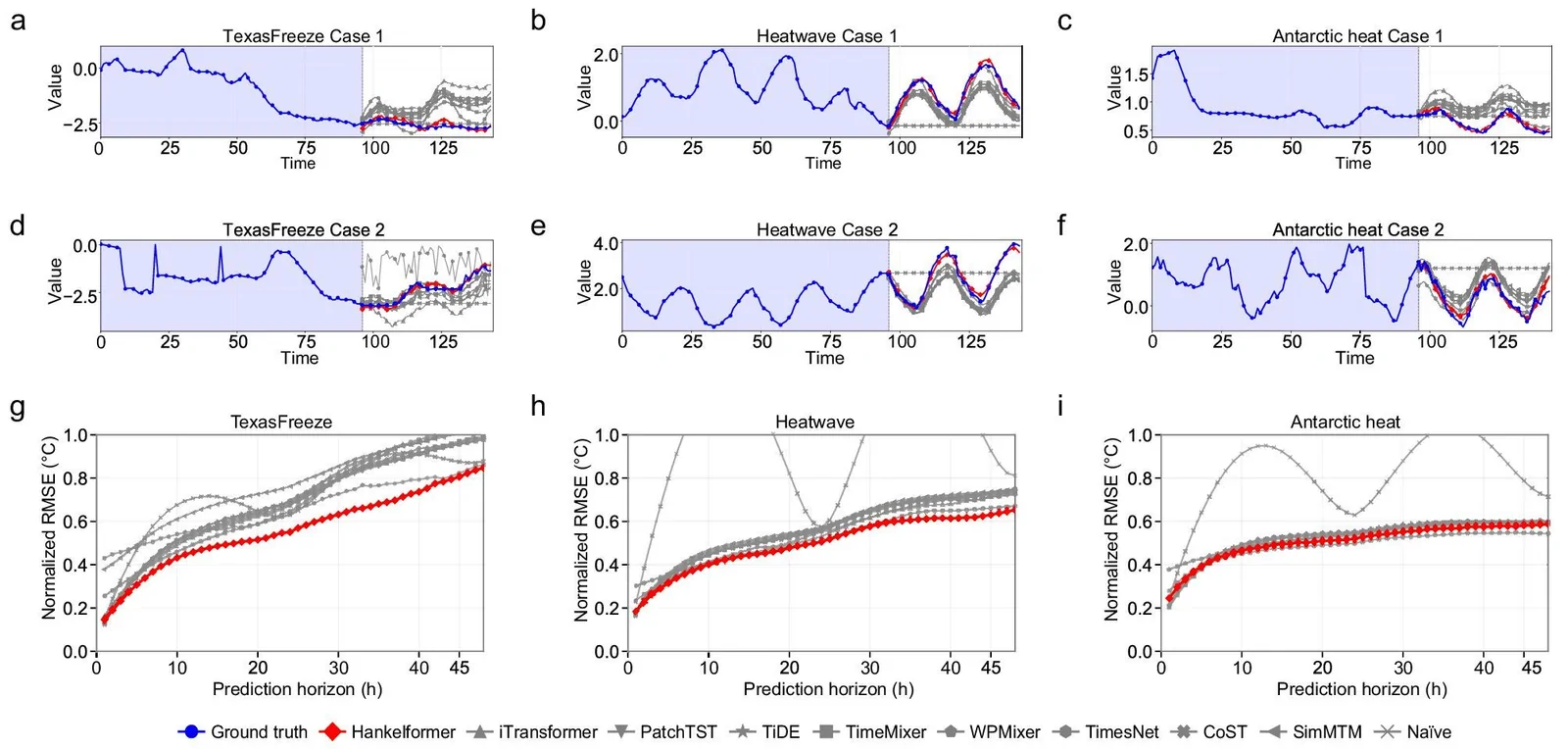
Comprehensive analysis of forecasting performance during extreme weather events. (a, d) Two qualitative case studies from the TexasFreeze dataset, showing a 48-hour temperature forecast. Hankelformer accurately captures the severe temperature drop, while baselines fail. (b, e) Two similar cases from the Heatwave dataset, where Hankelformer successfully predicts the sharp peak in temperature. (c, f) Two representative cases from the Antarctic Heat dataset. (g–i) Normalized root mean squared error as a function of prediction horizon for the TexasFreeze, Heatwave and Antarctic Heat datasets, respectively. Hankelformer maintains the lowest error across the entire forecast window. RMSE, root mean squared error.

In addition to the MSE and MAE metrics, to directly evaluate performance on extreme events, we further incorporated the Symmetric Extremal Dependence Index (SEDI) and the Frequency Bias Index (FBI) as additional evaluation metrics. SEDI is a metric specifically designed to evaluate the predictive performance of extreme weather events, where positive values indicate that the prediction outperforms random guess, and the closer the value is to 1, the more accurate the prediction. FBI is used to measure the discrepancy between the forecast frequency of an event and the actual observed frequency of the event; the closer the value is to 1, the greater the agreement between the forecast frequency and the observed frequency. For details on how these metrics are calculated, see the ‘Methods’ section. The results are summarized in Table 4. Overall, Hankelformer exhibits more consistent alignment with observed extreme-event amplitudes and frequencies across multiple forecasting horizons compared with the baseline models. The results indicate that Hankelformer excels not only in reducing average forecast error but also in specifically capturing the dynamics of extreme weather events.

**Table 4.** Extreme event forecasting performance measured by SEDI and FBI.​ Results are shown for multistep (12, 24, 48 and 96) hourly temperature prediction on the Antarctic Heat, Heatwave and TexasFreeze datasets. SEDI measures detection skill (higher is better). FBI measures frequency calibration (closer to 1 is better). The best SEDI and FBI scores for each horizon and dataset are highlighted in bold. The ‘Avg’ row reports the average score across all horizons. ‘1st Cnt’ indicates the number of times a model achieved the best score for a given metric.

		Hankelformer		WPMixer		iTransformer		TimeMixer		TiDE		PatchTST		SimMTM		CoST		TimesNet	
		(Ours)		(2025)		(2024)		(2024)		(2023)		(2023)		(2023)		(2022)		(2022)	
Dataset	Time step	SEDI	FBI	SEDI	FBI	SEDI	FBI	SEDI	FBI	SEDI	FBI	SEDI	FBI	SEDI	FBI	SEDI	FBI	SEDI	FBI
Antarctic Heat	12	0.715	0.793	0.720	0.777	0.719	0.759	**0.723**	0.746	0.714	0.778	**0.723**	0.658	0.683	0.610	0.688	0.630	0.705	**0.857**
	24	**0.699**	0.647	0.684	0.627	0.690	0.667	0.694	0.631	0.688	0.702	0.682	0.540	0.697	0.608	0.553	0.517	0.672	**0.712**
	48	**0.657**	**0.706**	0.637	0.625	0.634	0.002	0.638	0.527	0.635	0.629	0.621	0.518	0.577	0.680	0.562	0.506	0.616	0.585
	96	**0.595**	**0.641**	0.579	0.557	0.557	0.377	0.562	0.483	0.569	0.604	0.556	0.486	0.554	0.583	0.465	0.580	0.552	0.651
	Avg	**0.667**	0.697	0.655	0.647	0.650	0.451	0.654	0.597	0.652	0.678	0.646	0.551	0.628	0.620	0.567	0.558	0.636	**0.701**
Heatwave	12	0.695	**0.922**	0.729	0.683	0.716	0.837	0.778	0.691	0.792	0.674	**0.798**	0.595	0.413	0.661	0.693	0.860	0.727	0.855
	24	0.779	**0.808**	0.705	0.576	0.765	0.740	0.781	0.546	**0.782**	0.568	0.779	0.493	0.245	0.592	0.770	0.803	0.757	0.737
	48	**0.746**	**0.681**	0.617	0.430	0.737	-0.105	0.710	0.374	0.710	0.426	0.703	0.360	0.305	0.350	0.533	0.653	0.617	0.282
	96	**0.542**	**0.131**	0.459	0.078	0.468	0.055	0.477	0.056	0.467	0.066	0.438	0.049	0.298	0.127	0.019	0.096	0.457	0.028
	Avg	**0.691**	**0.636**	0.628	0.442	0.672	0.382	0.687	0.417	0.688	0.434	0.690	0.374	0.315	0.433	0.504	0.603	0.640	0.476
TexasFreeze	12	0.706	0.898	0.701	0.985	0.748	0.770	0.703	0.975	0.695	**0.993**	0.717	0.932	0.627	0.879	0.515	0.661	**0.756**	0.651
	24	0.725	0.842	0.697	1.013	0.741	0.715	0.698	**1.004**	0.690	1.014	0.715	0.934	0.691	0.826	0.309	0.636	**0.755**	0.638
	48	0.700	**1.023**	0.689	1.099	**0.721**	0.806	0.703	1.036	0.689	1.078	0.705	1.025	0.630	0.913	0.020	0.530	0.709	0.636
	96	**0.700**	1.071	0.671	1.239	0.673	**1.023**	0.686	1.086	0.668	1.212	0.686	1.081	0.686	0.908	0.020	0.643	0.681	0.867
	Avg	0.708	0.959	0.690	1.084	0.721	0.829	0.698	1.025	0.686	1.074	0.706	**0.993**	0.659	0.882	0.207	0.618	**0.725**	0.698
1st Cnt		**8**	**8**	0	0	1	1	1	1	1	1	2	1	0	0	0	0	3	3

### Ablation study

To evaluate the individual contributions of Hankel-AUG and the contrastive learning module, we conducted ablation studies on the full Hankelformer model using four representative datasets: 90D-Lorenz, ECL, Traffic and TexasFreeze, which span the dynamics, energy, transportation and extreme weather domains, respectively. Figure 5a–d presents the corresponding MSE and MAE results. Notably, introducing Hankel-AUG without the contrastive loss often degrades performance, resulting in even higher errors than the baseline. Neither module alone is sufficient for achieving optimal performance. The reason can be explained as follows. While Hankel-based augmentation increases representation diversity and exposes informative dynamical patterns, it also introduces a nontrivial distributional discrepancy between the original sequence representation and the augmented representation. Without the contrastive objective, the encoder has no explicit mechanism to align these heterogeneous representations in the latent space. Consequently, directly fusing the original and augmented features may introduce optimization conflicts and unstable feature interactions, which can negatively affect forecasting performance. From a representation learning perspective, the Hankel-AUG module increases structural diversity, while the contrastive loss regularizes the latent geometry to ensure consistent and robust representations.

**Figure 5.**
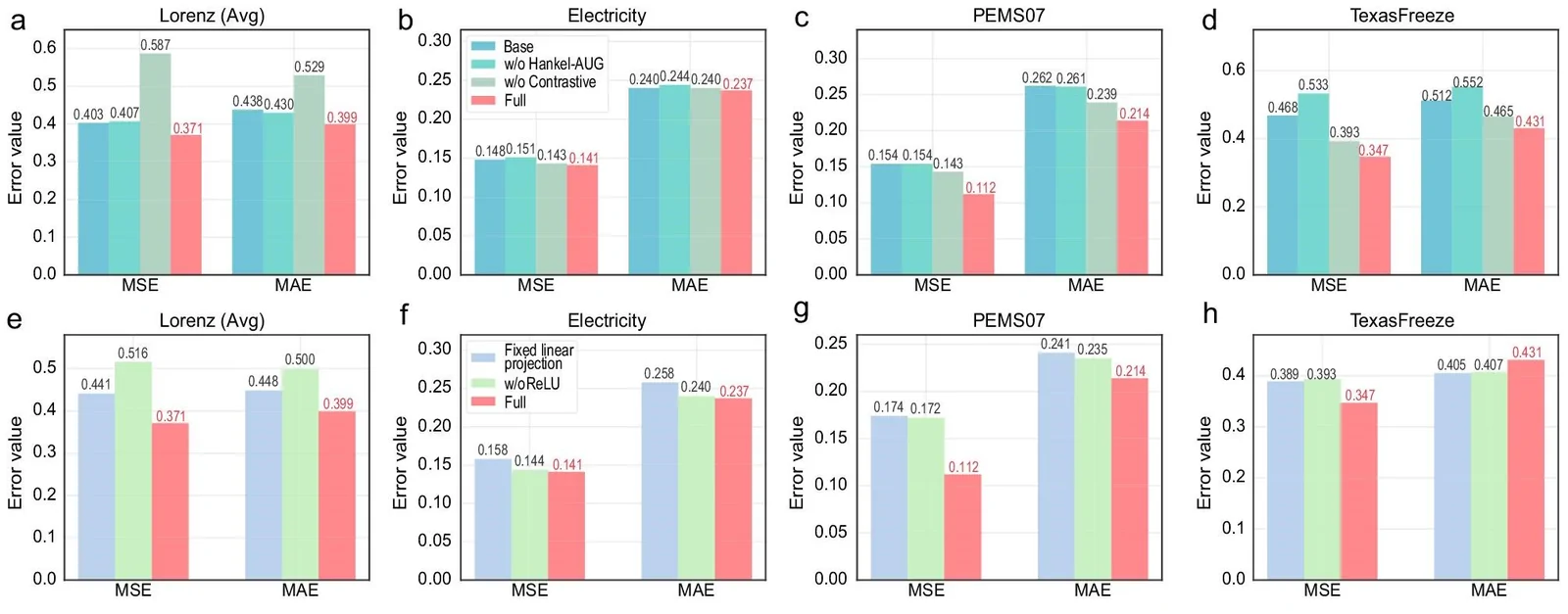
The performance of the full model is compared against variants without key components. (a–d) The effectiveness of the Hankel-AUG and contrastive loss modules on the Lorenz, Electricity, PEMS07, and TexasFreeze datasets, respectively. The consistent lower error of the full model highlights the significant contribution of both the Hankel-AUG modules, the contrastive learning objective and the learnable MLP layer in the Hankel-AUG module. (e–h) The effectiveness of replacing the MLP in the Hankel-AUG module with a fixed linear projection and of removing the ReLU activation function on the same datasets.

In addition, a learnable MLP has been incorporated into Hankel-AUG module. To empirically investigate the necessity and effect of this learnable component, we conducted an extended ablation study comparing three variants of the Hankel-AUG module: the full Hankelformer model with non-linear MLP projection, a learnable linear projection without non-linear activation and a fixed linear projection. As shown in the Fig. 5e–h (see also Tables S2 and S3), the full model consistently achieves the best performance. These results demonstrate that while the Hankel construction provides a critical structured starting point, the learnable non-linear transformation is essential for adapting this representation to achieve robustness and high accuracy in practical forecasting scenarios.

Finally, compared to existing explicit spatial dynamics modeling methods, our approach employs a dual explicit modeling, which are handled by the Hankel-AUG and Transformer encoder, respectively. Notably, the accuracy of spatiotemporal dynamics modeling depends on the proportion of Hankel-augmented variables. To validate this point, we conducted an ablation study where Hankel augmentation was applied to only a subset of input variables (0%, 25%, 50%, 75% and 100%). The results, presented in Table S4, show that forecast performance improves as the proportion of augmented variables increases, with the full model (100%) achieving the best overall results on average across all datasets and horizons.

## DISCUSSION

We introduced Hankelformer, a novel MTS forecasting architecture with the spatiotemporal representation, which achieves satisfactory performance through the integration of structured Hankel-AUG and end-to-end contrastive learning. Our approach addresses fundamental limitations in current deep learning methods, insufficient local spatiotemporal pattern extraction and limited robustness to distribution shifts, through mathematically principled innovations grounded in dynamical representation and information-theoretic principles, i.e. the delay embedding theorem and Hankel matrix decomposition. Comprehensive evaluation across nine benchmark datasets demonstrates state-of-the-art performance, with Hankelformer ranking first in almost all metric-horizon combinations. Notably, the architecture exhibits notable robustness in extreme temperature forecasting, achieving substantial improvements of up to 34% MSE reduction compared to leading baselines. The notable effectiveness during extreme weather events (Table 3 and Fig. 4) reveals fundamental insights into temporal pattern recognition under distribution shifts. The Hankel-based augmentation naturally captures local precursor patterns that often precede extreme transitions, while contrastive learning encourages the model to learn features invariant to such local perturbations. This combination enables prediction of unprecedented patterns by leveraging learned invariances rather than relying solely on historical precedent. Rigorous ablation studies confirm the necessity of each architectural component, while noise robustness analysis validates the intrinsic stability of learned representations. The success of structured augmentation principles suggests promising extensions to other time-series tasks including anomaly detection, classification and multimodal forecasting. The demonstrated robustness properties make Hankelformer particularly suitable for safety-critical applications in energy systems, transportation networks and climate monitoring where prediction failures can have severe consequences.

The strong performance of Hankelformer stems from three complementary design principles that address fundamental limitations in current time-series forecasting. First, the Hankel-AUG module leverages structured matrix decomposition theory to extract local spatiotemporal patterns while preserving temporal coherence—a critical advantage over generic augmentation strategies that often destroy sequential dependencies [47,48]. Unlike traditional sliding window approaches, Hankel matrices systematically capture all possible subsequence interactions within the specified window, providing a mathematically principled foundation for augmentation that aligns with classical signal processing theory [63,64]. Second, the contrastive learning framework operates on information-theoretic principles [49,50], maximizing mutual information between structured views to fuse the spatiotemporal representations while promoting invariant feature learning. Our noise robustness results (Table 1 and Fig. 2g) provide empirical validation that this approach yields representations inherently robust to stochastic perturbations—a crucial property for handling extreme events where historical distributions become unreliable. The graceful degradation (only 17% MSE increase across σ = 0–1.0) confirms that contrastive learning creates representations that capture essential temporal dynamics rather than spurious correlations. Third, the dual-stream architecture with explicit feature fusion ensures that robust representations learned through contrastive objectives directly contribute to forecasting performance, mitigating potential pretraining–finetuning mismatch that plagues separate pretraining approaches [56–62]. This end-to-end integration enables simultaneous optimization for both representation robustness and predictive accuracy.

Compared to state-of-the-art Transformer variants [33–43], Hankelformer’s unique advantage lies in explicitly addressing the local pattern extraction limitation through structured augmentation, which provides effective dynamic constraints for contrastive learning. While models like PatchTST [36] and iTransformer [37] excel at capturing global dependencies through refined attention mechanisms, they often miss critical local dynamics that serve as early warning signals for extreme events. Our ablation results (Fig. 5) demonstrate that replacing structured Hankel augmentation with simple noise degrades performance by 53.6%—worse than baseline—highlighting that naïve contrastive learning is insufficient. Relative to existing time-series contrastive learning methods (TS2Vec [58], CoST [59] and TF-C [60]), our approach distinguishes itself through: (i) mathematically motivated augmentation preserving temporal structure due to the delay embedding strategy, (ii) end-to-end integration mitigating potential pretraining–finetuning mismatch and (iii) explicit focus on local spatiotemporal pattern diversity. The consistent improvements across most metric-horizon combinations validate this principled approach.

Several limitations of this study warrant acknowledgment. First, the computational overhead associated with the dual-stream architecture increases training time by ∼1.3× compared to single-stream baselines, highlighting the need for future work on more efficient variants or adaptive augmentation strategies. For detailed metrics related to computational efficiency and comparisons with other benchmark methods, see Table S5. Second, the geographic scope of the extreme weather datasets is currently limited to limited regimes, validation across diverse climate regimes and geographical regions is necessary to strengthen claims regarding generalizability. Third, while the model demonstrates superior performance, its interpretability remains limited, similar to other existing works. Identifying which specific local patterns contribute to predictions of extreme events could enhance trustworthiness in high-stakes operational settings. Future research could incorporate attention visualization techniques or develop explainable variants of the Hankel-AUG mechanism. Fourth, our method is currently primarily applicable to extreme temperature events. For scenarios involving more rapid and abrupt changes, such as heavy rainfall events (see Fig. S2 and Table S6 for test results), more advanced theories and methods need to be introduced. Another promising direction is integrating graph neural networks [70,71] into Hankelformer to more explicitly incorporate geographical topology (e.g. 2D or 3D meteorological fields) and spatial connectivity, employing localized or sparse attention masks to restrict information exchange to physically relevant neighborhoods, and exploring hierarchical multiscale architectures for jointly modeling local and large-scale dynamics. Finally, hyperparameter sensitivity—particularly in the choice of Hankel window size *W* and contrastive loss weight *λ *—currently requires empirical tuning (see Fig. S3 and Tables S7–S9 for sensitivity analyses on several representative datasets). The development of adaptive or theoretically guided parameter selection strategies would improve practical applicability across varying temporal scales and application domains. Future work will focus on extending these principles to broader temporal analysis tasks, developing more efficient architectural variants and enhancing model interpretability for high-stakes applications. The integration of self-supervised learning with structured domain knowledge represents an important step toward more principled approaches in time-series modeling, moving beyond generic deep learning adaptations toward methods that respect the unique characteristics of temporal data. This work establishes a foundation for future research in robust temporal representation learning under real-world, non-stationary conditions.

## METHODS

Detailed methods are given in the online Supplementary data.

## Supplementary Material

nwag456_Supplemental_File

## Data Availability

The official implementation of the Hankelformer and the generation method of the 90D-Lorenz system datasets are available at https://github.com/qcyqcy/Hankelformer-new. Six publicly available datasets could be found at https://github.com/thuml/Time-Series-Library. The extreme weather event datasets could be found at https://github.com/taohan10200/WEATHER-5K.

## References

[1] Mashlakov A, Kuronen T, Lensu L et al. Assessing the performance of deep learning models for multivariate probabilistic energy forecasting. Appl Energy 2021; 285: 116405. 10.1016/j.apenergy.2020.116405

[2] Mystakidis A, Koukaras P, Tsalikidis N et al. Energy forecasting: a comprehensive review of techniques and technologies. Energies 2024; 17: 1662. 10.3390/en17071662

[3] Han L, Chen XY, Ye HJ et al. SOFTS: efficient multivariate time series forecasting with series-core fusion. In: Advances in Neural Information Processing Systems, Vol. 37. 2024, 64145–75. 10.52202/079017-2046

[4] Li W, Yang G, Xiong Z et al. A traffic flow prediction model based on dynamic graph convolution and adaptive spatial feature extraction. Symmetry 2025; 17: 1007.

[5] Chang A, Ji Y, Bie Y. Transformer-based short-term traffic forecasting model considering traffic spatiotemporal correlation. Front Neurorobot 2025; 19: 1527908. 10.3389/fnbot.2025.1527908 PMC11799296 39917631

[6] Li S, Yang W, Zhang P et al. ClimateLLM: efficient weather forecasting via frequency-aware large language models [preprint]. arXiv: 2502.11059.

[7] Chen S, Long G, Jiang J et al. Foundation models for weather and climate data understanding: a comprehensive survey [preprint]. arXiv: 2312.03014.

[8] Salman AG, Kanigoro B, Heryadi Y. Weather forecasting using deep learning techniques. In: International Conference on Advanced Computer Science and Information Systems 2015; 281–5.

[9] Nguyen-Thai B, Le V, Tieu NDT et al. Learning evolving relations for multivariate time series forecasting. Appl Intell 2024; 54: 3918–32.

[10] Bui C, Pham N, Vo A et al. Time series forecasting for healthcare diagnosis and prognostics with the focus on cardiovascular diseases. In: International conference on the development of biomedical engineering in Vietnam, Vol. 6, 2017; 809–18.

[11] Melaku ND, Fares A, Awal R. Exploring the impact of winter storm Uri on power outage, air quality, and water systems in Texas, USA. Sustainability 2023; 15: 4173. 10.3390/su15054173

[12] Busby JW, Baker K, Bazilian MD et al. Cascading risks: understanding the 2021 winter blackout in Texas. Energy Res Soc Sci 2021; 77: 102106. 10.1016/j.erss.2021.102106

[13] Lee CC, Maron M, Mostafavi A. Community-scale big data reveals disparate impacts of the Texas winter storm of 2021 and its managed power outage. Humanit Soc Sci Commun 2022; 9: 335. 10.1057/s41599-022-01353-8 PMC9510185 36187845

[14] Aderibigbe AO, Ani EC, Ohenhen PE et al. Enhancing energy efficiency with AI: a review of machine learning models in electricity demand forecasting. Eng Sci Technol J 2023; 4: 341–56. 10.51594/estj.v4i6.636

[15] Ghimire S, Deo RC, Casillas-Pérez D et al. Probabilistic-based electricity demand forecasting with hybrid convolutional neural network-extreme learning machine model. Eng Appl Artif Intell 2024; 132: 107918. 10.1016/j.engappai.2024.107918

[16] Chen J, Zheng L, Hu Y et al. Traffic flow matrix-based graph neural network with attention mechanism for traffic flow prediction. Inf Fusion 2024; 104: 102146. 10.1016/j.inffus.2023.102146

[17] Medina-Salgado B, Sánchez-DelaCruz E, Pozos-Parra P et al. Urban traffic flow prediction techniques: a review. Sustain Comput Inform 2022; 35: 100739.

[18] Camps-Valls G, Fernández-Torres MÁ, Cohrs KH et al. Artificial intelligence for modeling and understanding extreme weather and climate events. Nat Commun 2025; 16: 1919. 10.1038/s41467-025-56573-8 PMC11850610 39994190

[19] Materia S, García LP, van Straaten C et al. Artificial intelligence for climate prediction of extremes: state of the art, challenges, and future perspectives. WIREs Clim Change 2024; 15: e914. 10.1002/wcc.914

[20] Box GEP, Jenkins GM, Reinsel GC et al. Time Series Analysis: Forecasting and Control, 5th edn. Hoboken: John Wiley & Sons, 2015.

[21] Box GEP, Jenkins GM. Some recent advances in forecasting and control. Appl Stat 1968; 17: 91–109. 10.2307/2985674

[22] Sims CA . Macroeconomics and reality. Econometrica 1980; 48: 1–48. 10.2307/1912017

[23] Rady EHA, Fawzy H, Abdel Fattah AM. Time series forecasting using tree based methods. J Stat Appl Probab 2021; 10: 229–44.

[24] Kurani A, Doshi P, Vakharia A et al. A comprehensive comparative study of artificial neural network (ANN) and support vector machines (SVM) on stock forecasting. Ann Data Sci 2023; 10: 183–208. 10.1007/s40745-021-00344-x

[25] Xu K, Chen L, Patenaude JM et al. Kernel representation learning with dynamic regime discovery for time series forecasting. In: Pacific-Asia Conference on Knowledge Discovery and Data Mining. Springer, Singapore, 2024, 251–63. 10.1007/978-981-97-2266-2 10.1007/978-981-97-2266-2_20

[26] Elman JL . Finding structure in time. Cogn Sci 1990; 14: 179–211. 10.1207/s15516709cog1402_1

[27] Hochreiter S, Schmidhuber J. Long short-term memory. Neural Comput 1997; 9: 1735–80. 10.1162/neco.1997.9.8.1735 9377276

[28] Gers FA, Schmidhuber J, Cummins F. Learning to forget: continual prediction with LSTM. Neural Comput 2000; 12: 2451–71. 10.1162/089976600300015015 11032042

[29] Van Den Oord A, Dieleman S, Zen H et al. WaveNet: a generative model for raw audio [preprint]. arXiv: 1609.03499.

[30] Bai S, Kolter JZ, Koltun V. An empirical evaluation of generic convolutional and recurrent networks for sequence modeling [preprint]. arXiv: 1803.01271.

[31] Bahdanau D, Cho K, Bengio Y. Neural machine translation by jointly learning to align and translate [preprint]. arXiv: 1409.0473.

[32] Vaswani A, Shazeer N, Parmar N et al. Attention is all you need. In: Advances in Neural Information Processing Systems, Vol. 30. 2017, 5998–6008.

[33] Zhou H, Zhang S, Peng J et al. Informer: beyond efficient transformer for long sequence time-series forecasting. In: Proceedings of the AAAI Conference on Artificial Intelligence, Vol. 35. 2021, 11106–15. 10.1609/aaai.v35i12.17325

[34] Wu H, Xu J, Wang J et al. Autoformer: decomposition transformers with auto-correlation for long-term series forecasting. In: Advances in Neural Information Processing Systems, Vol. 34. 2021, 22419–30.

[35] Wu H, Hu T, Liu Y et al. TimesNet: temporal 2D-variation modeling for general time series analysis. In: International Conference on Learning Representations, Vol. 11. 2023, 1–23.

[36] Nie Y, Nguyen NH, Sinthong P et al. A time series is worth 64 words: long-term forecasting with transformers. In: International Conference on Learning Representations, Vol. 11. 2023, 1–8.

[37] Liu Y, Hu T, Zhang H et al. iTransformer: inverted transformers are effective for time series forecasting. In: International Conference on Learning Representations, Vol. 12. 2024, 1–24.

[38] Zhou T, Ma Z, Wen Q et al. FEDformer: frequency enhanced decomposed transformer for long-term series forecasting. In: International Conference on Machine Learning, Vol. 162. 2022, 27268–86.

[39] Woo G, Liu C, Sahoo D et al. ETSformer: exponential smoothing transformers for time-series forecasting. In: International Conference on Learning Representations, Vol. 11. 2023, 1–24.

[40] Liu S, Yu H, Liao C et al. Pyraformer: low-complexity pyramidal attention for long-range time series modeling and forecasting. In: International Conference on Learning Representations, Vol. 10. 2022, 1–20.

[41] Li S, Jin X, Xuan Y et al. Enhancing the locality and breaking the memory bottleneck of transformer on time series forecasting. In: Advances in Neural Information Processing Systems, Vol. 32. 2019, 5244–54.

[42] Kitaev N, Kaiser L, Levskaya A. Reformer: the efficient transformer. In: International Conference on Learning Representations, Vol. 8. 2020, 1–12.

[43] Zhou T, Ma Z, Wen Q et al. FiLM: frequency-improved Legendre memory model for long-term time series forecasting. In: Advances in Neural Information Processing Systems, Vol. 35. 2022, 12677–90. 10.52202/068431-0921

[44] Liu J, Shen Z, He Y et al. Towards out-of-distribution generalization: a survey [preprint]. arXiv: 2108.13624.

[45] Wang J, Lan C, Liu C et al. Generalizing to unseen domains: a survey on domain generalization. IEEE Trans Knowl Data Eng 2022; 35: 8052–72.

[46] Zhou K, Liu Z, Qiao Y et al. Domain generalization: a survey. IEEE Trans Pattern Anal Mach Intell 2023; 45: 4396–415. 10.1109/TPAMI.2022.3195549 35914036

[47] Iwana BK, Uchida S. An empirical survey of data augmentation for time series classification with neural networks. PLoS One 2021; 16: e0254841. 10.1371/journal.pone.0254841 PMC8282049 34264999

[48] Wen Q, Sun L, Yang F et al. Time series data augmentation for deep learning: a survey. In: Proceedings of the International Joint Conference on Artificial Intelligence, Vol. 13, 2021; 4653-60.

[49] Liu X, Zhang F, Hou Z et al. Self-supervised learning: generative or contrastive. IEEE Trans Knowl Data Eng 2023; 35: 857–76. 10.1109/TKDE.2021.3090866

[50] Balestriero R, Ibrahim M, Sobal V et al. A cookbook of self-supervised learning [preprint]. arXiv: 2304.12210.

[51] Van Den Oord A, Li Y, Vinyals O. Representation learning with contrastive predictive coding [preprint]. arXiv: 1807.03748.

[52] He K, Fan H, Wu Y et al. Momentum contrast for unsupervised visual representation learning. In: Proceedings of the IEEE/CVF Conference on Computer Vision and Pattern Recognition. Vol. 37. 2020, 9729–38.

[53] Chen T, Kornblith S, Norouzi M et al. A simple framework for contrastive learning of visual representations. In: International Conference on Machine Learning, Vol. 119. 2020, 1597–607.

[54] Devlin J, Chang MW, Lee K et al. BERT: pre-training of deep bidirectional transformers for language understanding [preprint]. arXiv: 1810.04805.

[55] Gao T, Yao X, Chen D. SimCSE: simple contrastive learning of sentence embeddings. In: Proceedings of the 2021 Conference on Empirical Methods in Natural Language Processing. 2021, 6894–910.

[56] Zhang K, Wen Q, Zhang C et al. Self-supervised learning for time series analysis: taxonomy, progress, and prospects. IEEE Trans Pattern Anal Mach Intell 2024; 46: 6775–94. 10.1109/TPAMI.2024.3387317 38598381

[57] Choi H, Kang P. Multi-task self-supervised time-series representation learning. Inf Sci 2024; 671: 120654. 10.1016/j.ins.2024.120654

[58] Yue Z, Wang Y, Duan J et al. TS2Vec: towards universal representation of time series. In: Proceedings of the AAAI Conference on Artificial Intelligence, Vol 36. 2022, 8980–7. 10.1609/aaai.v36i8.20881

[59] Woo G, Liu C, Sahoo D et al. CoST: contrastive learning of disentangled seasonal-trend representations for time series forecasting. In: International Conference on Learning Representations, Vol. 10, 2022, 1–10.

[60] Zhang X, Zhao Z, Tsiligkaridis T et al. Self-supervised contrastive pre-training for time series via time-frequency consistency. In: Advances in Neural Information Processing Systems, Vol. 35. 2022, 3988–4003. 10.52202/068431-0288

[61] Dong J, Wu H, Zhang H et al. SimMTM: a simple pre-training framework for masked time-series modeling. In: Advances in Neural Information Processing Systems, Vol. 36. 2023, 29996–30025. 10.52202/075280-1306

[62] Tonekaboni S, Eytan D, Goldenberg A. Unsupervised representation learning for time series with temporal neighborhood coding. In: International Conference on Learning Representations, Vol. 9. 2021, 1–10.

[63] Pintelon R, Schoukens J. System Identification: A Frequency Domain Approach. Hoboken: Wiley-IEEE Press, 2012. 10.1002/9781118287422

[64] Markovsky I, Willems JC, Van Huffel S et al. Exact and Approximate Modeling of Linear Systems: A Behavioral Approach. Philadelphia: SIAM, 2006. 10.1137/1.9780898718263

[65] Ma H, Leng S, Aihara K et al. Randomly distributed embedding making short-term high-dimensional data predictable. Proc Natl Acad Sci USA 2018; 115: E9994–10002. 10.1073/pnas.1802987115 PMC6205453 30297422

[66] Chen P, Liu R, Aihara K et al. Autoreservoir computing for multistep ahead prediction based on the spatiotemporal information transformation. Nat Commun 2020; 11: 4568. 10.1038/s41467-020-18381-0 PMC7486927 32917894

[67] Peng H, Chen P, Yang N et al. One-core neuron deep learning for time series prediction. Natl Sci Rev 2025; 12: nwae441. 10.1093/nsr/nwae441 PMC11737406 39830389

[68] Wu T, Gao X, An F et al. Predicting multiple observations in complex systems through low-dimensional embeddings. Nat Commun 2024; 15: 2242. 10.1038/s41467-024-46598-w PMC10933326 38472208

[69] Wang Z, Tao P, Chen L. Delayformer: spatiotemporal transformation for predicting high-dimensional dynamics. Adv Sci 2026; 13: e14671. 10.1002/advs.202514671 PMC12806438 41271574

[70] Oskarsson J, Landelius T, Deisenroth MP et al. Probabilistic weather forecasting with hierarchical graph neural networks. In: Advances in Neural Information Processing Systems, Vol. 37. 2024, 41577–648. 10.52202/079017-1316

[71] Gao Y, Wu H, Shu R et al. OneForecast: a universal framework for global and regional weather forecasting. In: International Conference on Machine Learning, Vol. 267. 2025, 18658–97.

