# Supplementary material for: Forecasting extreme temperature events by Hankel-augmented contrastive learning
Source: Natl Sci Rev. 2026 Jul 29;13(17):nwag456. doi: 10.1093/nsr/nwag456 (PMC13639192; doi:10.1093/nsr/nwag456)
Supplement: Supplementary file 1 [file nwag456_supplemental_file.pdf]

Methods

90D-Lorenz system

The synthetic 90D-Lorenz system dataset was generated as follows: First, we constructed a coupled Lorenz 90 system, which consists of 30 individual 3-dimensional Lorenz oscillators. The dynamics of each oscillator i (for $i = 1,2,\ldots,30$) are governed by the classic Lorenz equations, with a linear coupling term acting on the x variables between adjacent oscillators. The specific differential equations for oscillator i are given by:

$$\frac{dx_i}{dt} = \sigma(y_i - x_i) + c(x_{i+1} + x_{i-1} - 2x_i),$$
$$\frac{dy_i}{dt} = x_i(\rho - z_i) - y_i,$$
$$\frac{dz_i}{dt} = x_i y_i - \beta z_i,$$

where $\sigma = 10$, $\rho = 28$, and $\beta = 8/3$ are the standard Lorenz parameters. The coupling strength c was set to 0.3. The system's dynamical equations were solved using numerical integration with a time step of approximately 0.02, generating data for a total of 12,000 time points. To ensure that the time series data resides on the system's attractor, the initial 2,000 transient points were discarded. The subsequent 10,000 data points were retained as the clean, noise-free dataset. To simulate a range of real-world measurement conditions, a suite of noisy datasets was generated from the clean time series. Gaussian observational noise with a standard deviation of 1.0 was independently added to each variable in the clean dataset, resulting in 10 time series prediction datasets with different noise levels varied from 0.0 to 1.0 in increments 0.1 for evaluation.

Public datasets

Six publicly available datasets were employed to evaluate the general prediction performance of Hankelformer. Electricity (ECL): Hourly electricity consumption data records the electricity consumption every 1 hour from 321 customers (2012-2014), which totally contains 26,304 time points. Traffic: Hourly traffic occupancy data from 862 sensors in San Francisco Bay Area freeways (2015-2016), consists of 17,544 time

points. PEMS03/04/07/08: California traffic flow data from the Caltrans Performance Measurement System at 5-minute intervals. Specifically, PEMS03 comprises 26,208 time steps from 358 sensors; PEMS04 is composed of 16,992 time steps from 307 sensors; PEMS07 covers 28,224 time steps from 883 sensors; and PEMS08 includes 17,856 time steps from 170 sensors.

Custom extreme weather datasets

To evaluate robustness of Hankelformer under extreme weather conditions, three custom datasets were constructed from the National Centers for Environmental Information (NCEI) Integrated Surface Database. **TexasFreeze**: Hourly temperature data from 121 weather stations (or feature variables, i.e., $M=121$) across Texas, spanning January 1, 2017 00:00 to February 17, 2021 23:00. The dataset captures the unprecedented 2021 North American cold wave. **Heatwave**: Hourly temperature data from 70 weather stations in Oregon and Washington, spanning January 1, 2017 00:00 to June 30, 2021 23:00. The dataset captures the historic 2021 Pacific Northwest heat dome. **Antarctic heat**: Hourly temperature data from 22 weather stations near the Antarctic Peninsula during the anomalous warming event recorded on February 6, 2020.

Data preprocessing

For standard benchmarks, chronological splitting followed established practices (70% training, 10% validation, 20% testing). For extreme temperature datasets, the custom splits ensure that training data precedes the extreme events, validation data includes lead-up conditions, and test data focuses on the actual extreme event periods. Data splits for TexasFreeze: training (2017-01-01 00:00 to 2019-12-31 23:00), validation (2020-01-01 00:00 to 2021-01-31 23:00), testing (2021-02-05 00:00 to 2021-02-17 23:00). Data splits for Heatwave: training (2017-01-01 00:00 to 2019-12-31 23:00), validation (2020-01-01 00:00 to 2021-06-22 23:00), testing (2021-06-23 00:00 to 2021-06-30 23:00). Data splits for Antarctic Heat: training (2017-01-01 00:00 to 2018-12-31 23:00),

validation (2019-01-01 00:00 to 2019-12-31 23:00), testing (2020-01-01 00:00 to 2020-02-17 23:00). All time series were normalized using Z-score normalization prior to training. Missing values were handled through linear interpolation where necessary.

Implementation Details of Hankelformer

The Hankelformer architecture comprises five main components: an MLP-based input projection layer (MLP_{in}), an MLP-based Hankelization layer (MLP_{Hankel}), a Transformer-based encoder, an MLP-based fusion layer (MLP_{fuse}), and an MLP-based prediction layer (MLP_{pred}). The number of Transformer blocks within the encoder is varied from {2, 3, 4} for different experiment settings.

In detail, given an input of multivariate time series $\boldsymbol{X} \in \mathbb{R}^{B\times M\times L}$, where B denotes the batch size, M is the number of variables and L is the number of time points. The Hankelization module first creates an augment representation $\boldsymbol{X}^{Aug} \in \mathbb{R}^{B\times M\times L}$ from the original input $\boldsymbol{X}$. Denote a sample $\boldsymbol{X}_b \in \mathbb{R}^{M\times L}$ within the batch B, where $b = 1,2,\dots,B$, W is the window size sliding along the time dimension L. A block-Hankel matrix $\boldsymbol{H}_b \in \mathbb{R}^{MW\times(L-W+1)}$ is firstly created by multivariable delay embedding as:

$$\boldsymbol{H}_b = \begin{bmatrix} \mathbf{X}^1 & \mathbf{X}^2 & \cdots & \mathbf{X}^{L-W+1} \\ \mathbf{X}^2 & \mathbf{X}^3 & \cdots & \mathbf{X}^{L-W+2} \\ \vdots & \vdots & \ddots & \vdots \\ \mathbf{X}^W & \mathbf{X}^{W+1} & \cdots & \mathbf{X}^L \end{bmatrix} = \begin{bmatrix} x_1^1 & x_1^2 & \dots & x_1^{L-W+1} \\ \vdots & \vdots & \ddots & \vdots \\ x_M^1 & x_M^2 & \dots & x_M^{L-W+1} \\ x_1^2 & x_1^3 & \dots & x_1^{L-W+2} \\ \vdots & \vdots & \ddots & \vdots \\ x_M^2 & x_M^3 & \dots & x_M^{L-W+2} \\ \vdots & \vdots & \ddots & \vdots \\ x_1^W & x_1^{W+1} & \dots & x_1^L \\ \vdots & \vdots & \ddots & \vdots \\ x_M^W & x_M^{W+1} & \dots & x_M^L \end{bmatrix},$$

where $i = 1,2,\dots,M, j = 1,2,\dots,L$, x_i^j is the j-th time point of the variable i. It is then transposed as $\boldsymbol{H}_b{}^T \in \mathbb{R}^{(L-W+1)\times MW}$, and fed into an MLP-based Hankelization layer:

$$\boldsymbol{H}_b^{2\,T} = MLP_{Hankel}\left(\boldsymbol{H}_b{}^T\right),$$

where $\boldsymbol{H}_b^{2\,T} \in \mathbb{R}^{(L-W+1)\times M}$ is processed by padding and transposing into $\boldsymbol{X}_b^{Aug} \in$

$\mathbb{R}^{M\times L}$ to generate the final augmented view for sample b. All samples' augmented views are concatenated along the batch dimension to formulate the $\boldsymbol{X}^{Aug} \in \mathbb{R}^{B\times M\times L}$. Then, the original input $\boldsymbol{X}$ and its augmented view $\boldsymbol{X}^{Aug}$ are projected by the same input layer independently as:

$$\boldsymbol{X}_{emb} = MLP_{in}(\boldsymbol{X}), \boldsymbol{X}_{emb}^{Aug} = MLP_{in}(\boldsymbol{X}^{Aug}),$$

where $\boldsymbol{X}_{emb}$ and $\boldsymbol{X}_{emb}^{Aug} \in \mathbb{R}^{B\times M\times D}$, D denotes the embedding dimension. Subsequently, the original embedding and the augmented embedding are processed independently by the Transformer encoder. Within each Transformer block, the input first passes through a multi-head self-attention (MSA) mechanism followed by a feed-forward network (FFN). Given the input $\boldsymbol{X}_{emb}$ and $\boldsymbol{X}_{emb}^{Aug}$ to a Transformer block, respectively, the operations are:

$$\begin{aligned}
\boldsymbol{Z}_{in} &= \text{LayerNorm}\left(\boldsymbol{X}_{emb} + \text{MSA}(\boldsymbol{X}_{emb})\right), \\
\boldsymbol{Z}_{emb} &= \text{LayerNorm}\left(\boldsymbol{Z}_{in} + FFN(\boldsymbol{Z}_{in})\right), \\
\boldsymbol{Z}_{in}^{Aug} &= \text{LayerNorm}\left(\boldsymbol{X}_{emb}^{Aug} + MSA\left(\boldsymbol{X}_{emb}^{Aug}\right)\right), \\
\boldsymbol{Z}_{emb}^{Aug} &= \text{LayerNorm}\left(\boldsymbol{Z}_{in}^{Aug} + FFN\left(\boldsymbol{Z}_{in}^{Aug}\right)\right),
\end{aligned}$$

where $\boldsymbol{Z}_{emb}$ and $\boldsymbol{Z}_{emb}^{Aug} \in \mathbb{R}^{B\times M\times D}$ denote the final output of the Transformer block of the original time series and its Hankelization view, respectively. In detail, given an input of $\boldsymbol{X}_{emb}^{tmp} \in \{\boldsymbol{X}_{emb}, \boldsymbol{X}_{emb}^{Aug}\}$, the MSA module is denoted as follows:

$$\boldsymbol{Q}_i = \boldsymbol{X}_{emb}^{tmp}\boldsymbol{W}_i^Q, \boldsymbol{K}_i = \boldsymbol{X}_{emb}^{tmp}\boldsymbol{W}_i^K, \boldsymbol{V}_i = \boldsymbol{X}_{emb}^{tmp}\boldsymbol{W}_i^V,$$

where $\boldsymbol{W}_i^Q, \boldsymbol{W}_i^K \in \mathbb{R}^{D\times d_k}$ and $\boldsymbol{W}_i^V \in \mathbb{R}^{D\times d_v}$ are learnable weight matrices for the i-th head. The output of a single attention head is then calculated as:

$$\text{head}_i = \text{softmax}\left(\frac{\boldsymbol{Q}_i\boldsymbol{K}_i^T}{\sqrt{d_k}}\right)\boldsymbol{V}_i.$$

The outputs of all N_h heads are concatenated and passed through a final linear projection to produce the MSA output:

$$\text{MSA}\left(\boldsymbol{X}_{emb}^{tmp}\right) = \text{Concat}\left(\text{head}_1, \text{head}_2, \ldots, \text{head}_{N_h}\right)\boldsymbol{W}^{out},$$

where $\boldsymbol{W}^{out}$ is another learnable weight matrix. Concurrently, the two representations

of the original and augmented views $\boldsymbol{Z}_{emb}$ and $\boldsymbol{Z}_{emb}^{Aug}$ are concatenated as

$$\boldsymbol{Z}_{emb}^{concat} = \text{Concat}(\boldsymbol{Z}_{emb}, \boldsymbol{Z}_{emb}^{Aug}).$$

The concatenated embeddings $\boldsymbol{Z}_{emb}^{concat} \in \mathbb{R}^{B\times M\times 2D}$ are fused into the embedding dimension $\boldsymbol{D}$ through an linear fusion layer as

$$\boldsymbol{Z}_{emb}^{fuse} = MLP_{\text{fuse}}\,(\boldsymbol{Z}_{emb}^{concat}), \boldsymbol{Z}_{emb}^{fuse} \in \mathbb{R}^{B\times M\times D}.$$

Finally, the fused feature representation is passed to the prediction layer, which maps it back to the original data space to generate the prediction $\widehat{\boldsymbol{Y}}$:

$$\widehat{\boldsymbol{Y}} = MLP_{\text{pred}}\,(\boldsymbol{Z}_{emb}^{fuse}), \widehat{\boldsymbol{Y}} \in \mathbb{R}^{B\times M\times H},$$

where H is the predict length.

Training and testing

All experiments were conducted with the Adam optimizer and early-stopping: training terminates if the validation error does not improve for three consecutive epochs. The max number of training epochs was set to 20. The number of attention heads was fixed to 8 and the temperature of the contrastive loss was set to $\tau = 0.5$. Remaining hyper-parameters were chosen by a light-weight grid search whose ranges are: Contrastive loss weight λ: 0.00005–0.375; batch size: {16, 32, 64, 128}; encoder layers: {2, 3, 4}; encoder dimension: {512, 1024}; feed-forward network dimension: {128, 512}; learning rate: $\{1 \times 10^{-4}, 3 \times 10^{-4}, 5 \times 10^{-4}, 1 \times 10^{-3}, 1.4 \times 10^{-3}, 1.2 \times 10^{-3}\}$; Hankel window size: 1-32. Among these hyperparameters, W typically has the greatest impact on prediction results because, in theory, the optimal W corresponds to the minimum number of variables needed to describe the system's state, which in our context relates to the key physical processes governing temperature extremes (e.g., advection, radiation). Although empirical methods (such as the false nearest neighbor (FNN[71]) method) exist for determining the value of W, experimental results show that the optimal W obtained by FNN performs worse in terms of prediction accuracy than the optimal W obtained via grid search (Fig. S4 and Table S10 for specific results). Furthermore, given that we typically use 96 time points as input, the maximum value

of W in the grid search does not exceed 32. It should be noted that while the optimal W value obtained using grid search demonstrates excellent predictive performance, it may not have a straightforward physical interpretation. Deeper mechanistic insights linking W to specific physical processes remain a valuable direction for future research. For complete parameter settings, please refer to Note 3 and Tables S11-S13.

The model was implemented in PyTorch and trained on NVIDIA RTX-3080Ti GPUs. Mean absolute error (MAE) and mean squared error (MSE) were applied to evaluate the predictive performance of Hankelformer and baselines:

$$\mathrm{MSE} = \frac{1}{NMH}\sum_{i=1}^{N}\sum_{j=1}^{M}\sum_{t=1}^{H}\left(\boldsymbol{Y}_{i,j,t} - \widehat{\boldsymbol{Y}}_{i,j,t}\right)^2,$$

$$\mathrm{MAE} = \frac{1}{NMH}\sum_{i=1}^{N}\sum_{j=1}^{M}\sum_{t=1}^{H}\left|\boldsymbol{Y}_{i,j,t} - \widehat{\boldsymbol{Y}}_{i,j,t}\right|,$$

where N represents the number of samples in the test set, H is the prediction horizon (the number of future time steps), and M denotes the number of variables.

In addition, to directly evaluate performance on extreme events, we incorporated the Symmetric Extremal Dependence Index (SEDI) and the Frequency Bias Index (FBI) as additional evaluation metrics. Following standard practice, extreme events for the temperature forecasts were defined using the 95th percentile threshold computed from the true observations of the respective datasets. We then calculated the number of hits or true positives (N_H), the number of false alarms or false positives (N_F), the number of misses or false negatives (N_M), and the number of true negatives (N_C). The definition of SEDI is

$$\mathrm{SEDI} = \frac{\ln(POFD) - \ln(POD) - \ln(1 - POFD) + \ln(1 - POD)}{\ln(POFD) + \ln(POD) + \ln(1 - POFD) + \ln(1 - POD)},$$

where probability of detection $POD = N_H/(N_H + N_M)$ and probability of false detection $\mathrm{POFD} = N_F/(N_F + N_C)$. The SEDI ranges from $(-\infty, 1]$, where a value of 1 indicates perfect ability to distinguish whether an extreme event will occur, 0 indicates no better than random guessing, and a negative value indicates worse performance than random guessing.The definition of FBI is

$$FBI = \frac{N_H + N_F}{N_H + N_M}.$$

The FBI ranges from $[0, +\infty)$, where a value of 1 indicates perfect calibration, a value less than 1 indicates underprediction, and a value greater than 1 indicates overprediction.

Note 1. Delay embedding and topological equivalence

For a general discrete-time M-dimensional dynamical system with evolution function $\phi: \mathbb{R}^M \to \mathbb{R}^M$, the dynamics can be written as

$$\mathbf{X}^{t+1} = \phi(\mathbf{X}^t),$$

where $\mathbf{X}^t = [x_1^t, x_2^t, \ldots, x_M^t]^T$ (T stands a transpose) represents the M-dimensional observation vector/state at time point $t = 1,2,\ldots,L$, sampled at equally time intervals from the system. The Takens' embedding theorem[1] is stated as:

If $\mathcal{M} \subseteq \mathbb{R}^M$ is an attractor with the box-counting dimension d, for a smooth diffeomorphism $\phi: \mathcal{M} \to \mathcal{M}$ and a smooth function $h: \mathcal{M} \to \mathbb{R}$, the mapping $\mathbf{\Phi}_{\phi,h}: \mathcal{M} \to \mathbb{R}^W$ is an embedding when $W > 2d$, that is

$$\begin{aligned}\mathbf{\Phi}_{\phi,h}(\mathbf{X}^t) &= [h(\mathbf{X}^{t-W+1}), h \circ \phi(\mathbf{X}^{t-W+1}), \cdots, h \circ \phi^{W-1}(\mathbf{X}^{t-W+1})]^T \\ &= [h(\mathbf{X}^{t-W+1}), h(\mathbf{X}^{t-W+2}), \cdots, h(\mathbf{X}^t)]^T,\end{aligned}$$

where symbol " $\circ$ " represents the function composition operation.

Letting $\mathbf{\Phi}_{\phi,h} = \mathbf{\Phi}_i$ for short and $h(\mathbf{X}^t)$ to be any target variable x_i^t, i.e., $h(\mathbf{X}^t) = x_i^t, i = 1,2,\ldots,M$, then we have the spatiotemporal information (STI) equation:

$$\mathbf{\Phi}_i(\mathbf{X}^t) = \left(x_i^{t-W+1}, x_i^{t-W+2}, \cdots, x_i^t\right)^T = \mathbf{Y}^t,$$

or the matrix form

$$\mathbf{\Phi}_i\left(\begin{bmatrix} x_1^W & x_1^{W+1} & \cdots & x_1^L \\ x_2^W & x_2^{W+1} & \cdots & x_2^L \\ \vdots & \vdots & \ddots & \vdots \\ x_M^W & x_M^{W+1} & \cdots & x_M^L \end{bmatrix}\right) = \begin{bmatrix} x_i^1 & x_i^2 & \cdots & x_i^{L-M+1} \\ x_i^2 & x_i^3 & \cdots & x_i^{L-M+2} \\ \vdots & \vdots & \ddots & \vdots \\ x_i^W & x_i^{W+1} & \cdots & x_i^L \end{bmatrix}.$$

As can be seen, $\mathbf{\Phi}_i$ maps the original observation state matrix $\mathbf{X}^{Cut} = \{\mathbf{X}^t\}_{t=W}^L$ to the delayed embedding Hankel matrix $\mathbf{H}_i = \{\mathbf{Y}^t\}_{t=W}^L$ for variable x_i^t. Since $\mathbf{\Phi}_i$ is a one-to-one mapping, $\mathbf{X}^{Cut}$ and $\mathbf{H}_i$ are topologically equivalent when $W > 2d$.

The multivariate topological equivalence is exactly the same kind of conjugacy/ diffeomorphism as in single-variable delayed embedding[2], but with delay coordinates built from vector observations, that is

$$\mathbf{\Phi}\left(\begin{bmatrix} x_1^W & x_1^{W+1} & \cdots & x_1^L \\ x_2^W & x_2^{W+1} & \cdots & x_2^L \\ \vdots & \vdots & \ddots & \vdots \\ x_M^W & x_M^{W+1} & \cdots & x_M^L \end{bmatrix}\right) = \begin{bmatrix} \mathbf{X}^1 & \mathbf{X}^2 & \cdots & \mathbf{X}^{L-W+1} \\ \mathbf{X}^2 & \mathbf{X}^3 & \cdots & \mathbf{X}^{L-W+2} \\ \vdots & \vdots & \ddots & \vdots \\ \mathbf{X}^W & \mathbf{X}^{W+1} & \cdots & \mathbf{X}^L \end{bmatrix} = \begin{bmatrix} x_1^1 & x_1^2 & \ldots & x_1^{L-W+1} \\ \vdots & \vdots & \ddots & \vdots \\ x_M^1 & x_M^2 & \ldots & x_M^{L-W+1} \\ x_1^2 & x_1^3 & \ldots & x_1^{L-W+2} \\ \vdots & \vdots & \ddots & \vdots \\ x_M^2 & x_M^3 & \ldots & x_M^{L-W+2} \\ \vdots & \vdots & \ddots & \vdots \\ x_1^W & x_1^{W+1} & \ldots & x_1^L \\ \vdots & \vdots & \ddots & \vdots \\ x_M^W & x_M^{W+1} & \ldots & x_M^L \end{bmatrix},$$

and also its reverse mapping, where the matrix on the right side is the multivariate delayed embedding matrix (or block Hankel matrix) $\mathbf{H} \in \mathbb{R}^{MW \times (L-W+1)}$. Similarly, $\mathbf{X}^{Cut}$ and $\mathbf{H}$ are topologically equivalent when $MW > 2d$.

In fact, in the Hankel-AUG module, for a sample $X_b \in \mathbb{R}^{M \times L}$ in a batch, the first step is to

construct the corresponding multivariate delayed embedding matrix $\boldsymbol{H}_b \in \mathbb{R}^{MW\times(L-W+1)}$. As shown by the mathematical proof above, $\boldsymbol{H}_b$ and a subset of sample $\boldsymbol{X}_b^{Cut}$ have a one-to-one correspondence in terms of states/vectors, meaning they are topologically equivalent. However, since $\boldsymbol{H}_b$ and the original input $\boldsymbol{X}_b$ have different dimensions, they cannot be directly applied for contrastive learning. Therefore, we need to use a mapping $\boldsymbol{\Psi}: \mathbb{R}^{MW} \to \mathbb{R}^{W}$ to perform feature embedding on $\boldsymbol{H}_b$ (reducing the dimension from *MW* to *M*) to obtain $\boldsymbol{H}_b^2 \in \mathbb{R}^{W\times(L-W+1)}$ while preserving the topological equivalence between the two, that is,

$$\boldsymbol{\Psi}(\boldsymbol{H}_b) = \boldsymbol{H}_b^2$$

or

$$\boldsymbol{\Psi}\left(\begin{bmatrix} x_1^1 & x_1^2 & \dots & x_1^{L-W+1} \\ \vdots & \vdots & \ddots & \vdots \\ x_M^1 & x_M^2 & \dots & x_M^{L-W+1} \\ x_1^2 & x_1^3 & \dots & x_1^{L-W+2} \\ \vdots & \vdots & \ddots & \vdots \\ x_M^2 & x_M^3 & \dots & x_M^{L-W+2} \\ \vdots & \vdots & \ddots & \vdots \\ x_1^W & x_1^{W+1} & \dots & x_1^L \\ \vdots & \vdots & \ddots & \vdots \\ x_M^W & x_M^{W+1} & \dots & x_M^L \end{bmatrix}\right) = \begin{bmatrix} h_1^W & h_1^{W+1} & \cdots & h_1^L \\ h_2^W & h_2^{W+1} & \cdots & h_2^L \\ \vdots & \vdots & \ddots & \vdots \\ h_M^W & h_M^{W+1} & \cdots & h_M^L \end{bmatrix}.$$

and also its reverse mapping. In practical applications, we use an MLP to approximate $\boldsymbol{\Psi}$. It should be noted that this learnable MLP is not trained in isolation, but rather trained end-to-end in conjunction with the contrastive learning module. Due to the constraints imposed by the contrastive loss, such a transformation from one vector to another performed by the MLP still preserves the topological dynamics of $\boldsymbol{H}_b$ in terms of states. Of course, due to the approximative or computational nature of the neural network, the topological dynamics of $\boldsymbol{H}_b$ and $\boldsymbol{H}_b^2$ are approximately equivalent, the degree of approximation depends on the properties of the MLP (number of parameters, activation functions, etc.). Finally, to obtain the augmented view $\boldsymbol{X}_b^{Aug} \in \mathbb{R}^{M\times L}$ with the same dimension as $\boldsymbol{X}_b$, we pad the time dimension from *L-W+1* to L. Clearly, this operation does not alter the topological equivalence between the $\boldsymbol{H}_b^2$ component in the augmented view and $\boldsymbol{H}_b$. In summary, under general conditions (smooth ϕ and h, embedding dimension $MW > 2d$), from a theoretical viewpoint the augmented view or state/vector constructed by the Hankel-AUG module is topologically equivalent to the original one. But taking into account real-world scenarios such as noise and non-static conditions, from a computational viewpoint, it is considered to be approximately equivalent to the original one in terms of states, that is,

$$\boldsymbol{\Psi\Phi}(\boldsymbol{X}_b^{Cut}) = \boldsymbol{H}_b^2,$$

or

$$\boldsymbol{\Psi\Phi}\left(\begin{bmatrix} x_1^W & x_1^{W+1} & \cdots & x_1^L \\ x_2^W & x_2^{W+1} & \cdots & x_2^L \\ \vdots & \vdots & \ddots & \vdots \\ x_M^W & x_M^{W+1} & \cdots & x_M^L \end{bmatrix}\right) = \begin{bmatrix} h_1^W & h_1^{W+1} & \cdots & h_1^L \\ h_2^W & h_2^{W+1} & \cdots & h_2^L \\ \vdots & \vdots & \ddots & \vdots \\ h_M^W & h_M^{W+1} & \cdots & h_M^L \end{bmatrix},$$

where $\boldsymbol{\Phi}$ is exact, while Ψ is approximated using a trainable MLP.

[1] F. Takens, in Dynamical Systems and Turbulence, Warwick 1980: proceedings of a symposium held at the University of Warwick 1979/80 Springer, 2006, 366-381.
[2] T. Sauer, J. A. Yorke, M. Casdagli, Journal of statistical Physics 1991, 65 (3), 579.

Note 2. Comparison of Hankelformer with existing contrastive learning methods and delayed embedding methods

From the perspective of contrastive learning, Hankelformer treats an instance within a batch and its augmented view as positive samples, while other instances in the same batch serve as negative samples, which is analogous to SimCLR[1] in computer vision or SimCSE[2] in natural language processing. However, whereas SimCLR augments images through rotation and cropping, and SimCSE augments sentences via random dropout, Hankelformer's augmentation strategy applies delay embedding to time series. The fundamental distinction among these three methods is that their data augmentation approaches are tailored to the characteristics of their respective modalities. Specifically, Hankelformer proves effective for time series data because cropping or dropout strategies from SimCLR/SimCSE would disrupt the dynamical continuity inherent in temporal data, whereas the Hankel-AUG-based augmentation strategy not only preserves such dynamics but also maintains the dynamical topological properties of the original system to a certain extent.

Furthermore, although several works have explored time series forecasting based on delay embedding/Hankel matrices, most of them construct a mapping between the original system and the embedded system through delay embedding, achieving prediction by learning this mapping from observed data. For instance, Wu et al.[3] embedded the topological features of high-dimensional systems into low-dimensional spaces based on delay embedding and manifold embedding theories, constructing a mapping from the feature manifold to the reconstructed manifold to enable multi-step prediction of all variables within complex systems. Wang et al.[4] treated delay-embedded Hankel matrices as two-dimensional images, employing a shared Vision Transformer (ViT) encoder to extract common dynamic representations across all variables, followed by independent linear decoders to predict all original variables in parallel. Nevertheless, to our knowledge, utilizing Hankel matrices for augmented representation of time series in conjunction with contrastive learning remains unexplored, constituting one of the core innovations of Hankelformer.

[1] Chen T, Kornblith S, Norouzi M et al. A simple framework for contrastive learning of visual representations. In: International conference on machine learning, 2020, 1597-1607.

[2] Gao T, Yao X and Chen D. Simcse: Simple contrastive learning of sentence embeddings. In: Proceedings of the 2021 conference on empirical methods in natural language processing, 2021, 6894-6910.

[3] Wu T, Gao X, An F et al. Predicting multiple observations in complex systems through low-dimensional embeddings. Nat Commun. 2024; 15: 2242.

[4] Wang Z, Tao P and Chen L. Delayformer: Spatiotemporal Transformation for Predicting High-Dimensional Dynamics. Advanced Science. 2025; 13: e14671.

Note 3. Baseline methods introduction

To provide a comprehensive and rigorous evaluation of Hankelformer, we compare its performance against representative baseline models, which are selected to encompass the primary architectural paradigms currently dominant in multivariate time series forecasting:

1. Transformer-based models: iTransformer and PatchTST, which represents the latest advancements in leveraging Transformer-based architectures for variate dependent and independent encoding paradigms, respectively.
2. MLP-based models: TiDE and TimeMixer, which serves as strong forecasting methods focusing on efficient temporal mapping through linear encoding or multi-scale mixing paradigms.
3. Hybrid Approaches: TimesNet and WPMixer, both adopting the multi-scale temporal mixing paradigm, represent the latest SOTA models for multivariate time series forecasting.
4. Pretrain-finetune Models: CoST and SimMTM, which both applying self-supervised pretraining and then finetuning on the forecasting tasks, serving as two-stage forecasting models.
5. Lower-bound Baseline: A naïve persistence baseline where all values are predicted using the final observed temporal point.

We achieved iTransformer, PatchTST, TimesNet, TimeMixer, TiDE, and WPMixer based on the open-sourced Time-Series-Library (https://github.com/thuml/Time-Series-Library) on the Github. CoST (https://github.com/salesforce/CoST) and SimMTM (https://github.com/thuml/SimMTM) were reproduced based on their own repositories. The naïve baseline was achieved by our own. Furthermore, to ensure a strictly fair comparison and eliminate potential biases from uneven tuning, all baseline models were evaluated using identical historical input windows (L=96) and forecasting horizons (H). The hyperparameters for all competing models were systematically optimized via grid search over comparable parameter ranges and search budgets under the same computing environment, ensuring that the reported performance gains of Hankelformer are strictly attributable to its architectural innovations rather than preferential hyperparameter tuning. For each model, we report the best result from reproduced five random seeds.

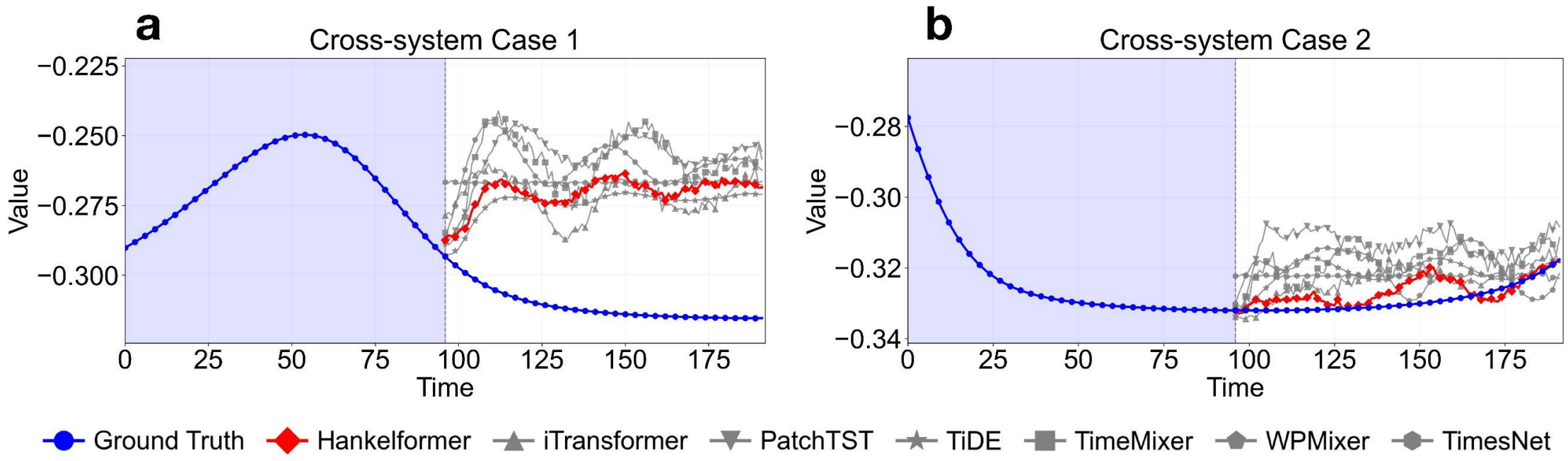

Fig. S1. Two representative forecast examples from the cross-system validation experiment.

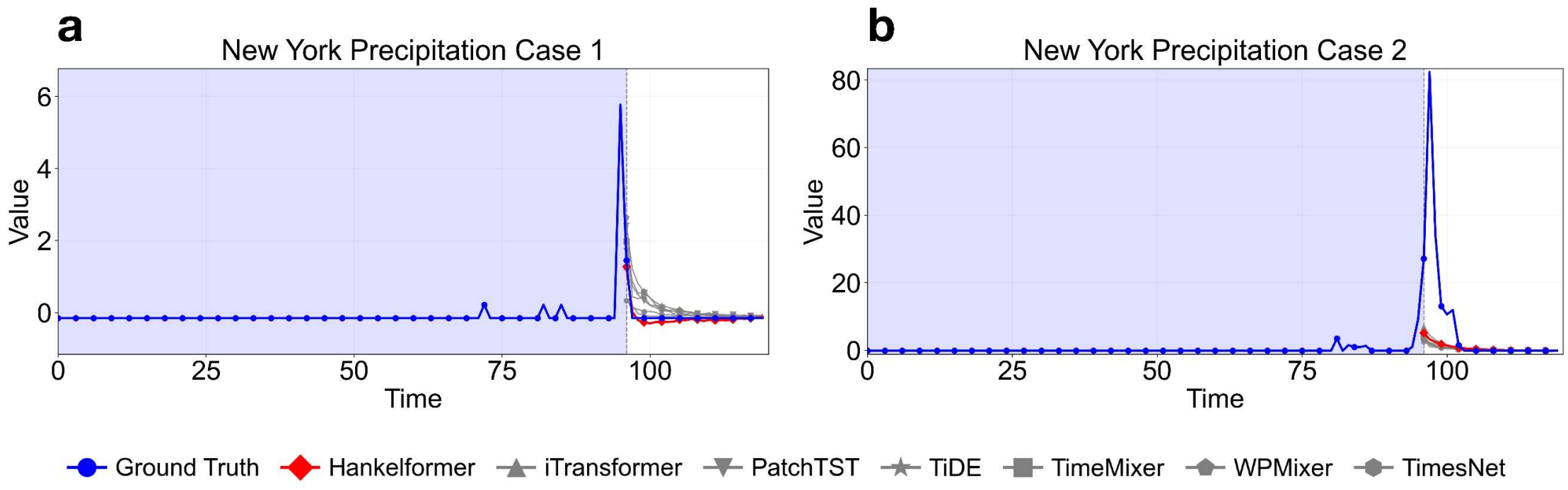

Fig. S2. Two representative forecast examples from the New York Precipitation dataset. The hourly liquid precipitation data was collected from 43 weather stations within a 1.5° (~170 km) radius around New York City, spanning from 00:00 on January 1, 2015, to 23:00 on December 31, 2021. All selected stations maintain complete hourly observations throughout the study period. Sourced from the NOAA NCEI Global Historical Climatology Network-hourly (GHCNh) dataset, with raw measurements collected by the Automated Surface Observing System (ASOS), the dataset was processed from 9-hour running accumulated precipitation records and covers multiple extreme heavy rainfall events across the New York metropolitan area. The data splits for the New York Precipitation dataset are as follows: training (January 1, 2015, 00:00 to December 31, 2019, 23:00), validation (January 1, 2020, 00:00 to August 31, 2021, 23:00), and testing (September 1, 2021, 00:00 to September 30, 2021, 23:00).

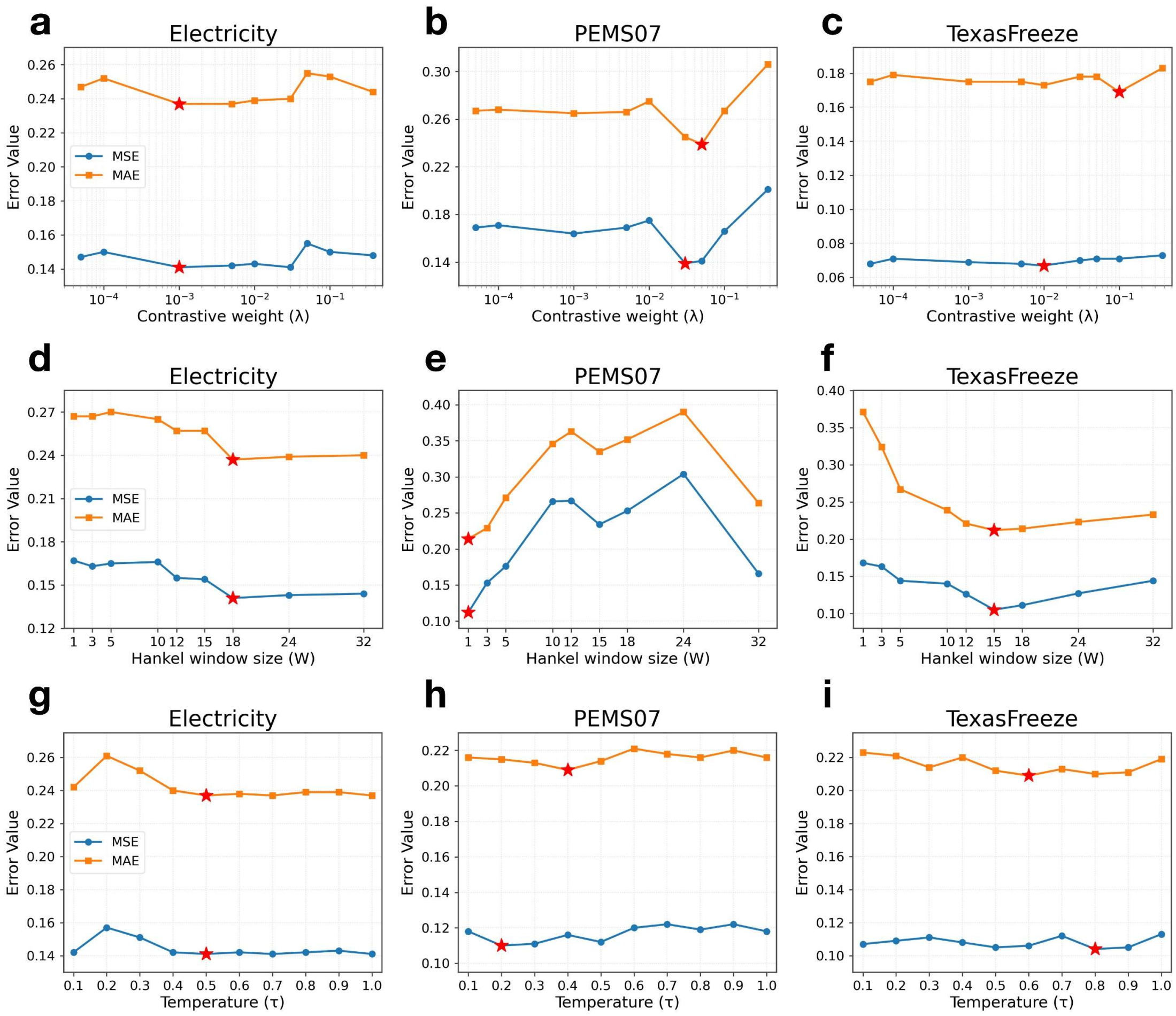

Fig. S3. Sensitivity analysis on key parameters. **a-c,** Sensitivity analysis on contrastive weight λ. **d-f,** Sensitivity analysis on Hankel window size W. **g-i,** Sensitivity analysis on the contrastive temperature τ. Best results are marked by red stars.

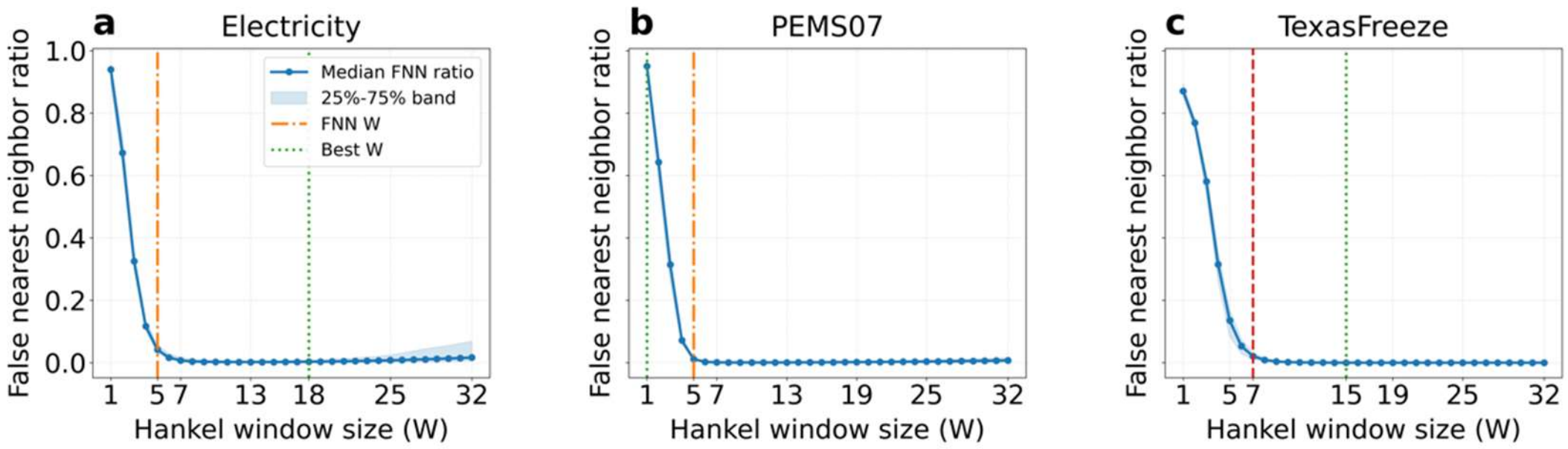

Fig. S4. Selection on Hankel window size *W*. "FNN *W*" denotes the best parameter detected by False nearest neighbor algorithm. "Best W" is the optimal parameter selected by a grid search on the validation set.

Table S1. Forecasting results on cross-system datasets. All predictions are conducted based on past 96 time points, where all models are trained on the Lorenz system data and evaluated on the Rössler system. The best performance is highlighted in bold.

Models	Rössler MSE
Hankelformer	**1.714**
iTransformer	2.714
PatchTST	1.985
WPMixer	2.010
TimeMixer	1.745
TiDE	4.054
TimesNet	2.053

Table S2. Ablation study on the Lorenz-90D dataset. All predictions are conducted based on past 96 time points and the prediction window is fixed at 96 steps. MSE and MAE for different models under increasing levels of additive Gaussian noise (σ). Best results are in bold.

Ablation	Hankelformer (OURS)		Base		w/o Hankel-AUG		w/o Contrastive		Fixed Linear Projection		w/o ReLU	
Noise Level	MSE	MAE	MSE	MAE	MSE	MAE	MSE	MAE	MSE	MAE	MSE	MAE
0	**0.348**	**0.380**	0.371	0.398	0.390	0.416	0.585	0.521	0.374	0.399	0.375	0.403
0.1	**0.353**	**0.384**	0.386	0.423	0.392	0.417	0.583	0.521	0.447	0.450	0.586	0.545
0.2	**0.348**	**0.381**	0.390	0.426	0.402	0.442	0.557	0.507	0.447	0.451	0.520	0.503
0.3	**0.356**	**0.386**	0.392	0.428	0.397	0.421	0.561	0.511	0.432	0.439	0.512	0.497
0.4	**0.367**	**0.396**	0.399	0.433	0.391	0.417	0.585	0.526	0.459	0.460	0.479	0.474
0.5	**0.374**	**0.401**	0.403	0.438	0.405	0.426	0.588	0.529	0.451	0.455	0.498	0.490
0.6	**0.369**	**0.398**	0.410	0.445	0.411	0.430	0.595	0.532	0.448	0.452	0.511	0.498
0.7	**0.373**	**0.402**	0.411	0.447	0.419	0.437	0.605	0.538	0.460	0.461	0.610	0.561
0.8	**0.393**	**0.417**	0.419	0.453	0.421	0.439	0.593	0.531	0.466	0.467	0.509	0.497
0.9	**0.393**	**0.418**	0.424	0.459	0.415	0.439	0.581	0.528	0.450	0.456	0.645	0.587
1.0	**0.407**	**0.428**	0.431	0.465	0.430	0.448	0.621	0.574	0.418	0.435	0.427	0.444

Table S3. Ablation results on the Hankel-AUG module. Forecasting performance (MSE/MAE) for multi-step forecasting across various horizons. Predictions are based on 96 historical time steps. Best results for each metric and horizon are highlighted in bold. The "Avg" row reports the average score across all horizons.

Ablation		Hankelformer (FULL)		Fixed Linear Projection		w/o ReLU	
Metric		MSE	MAE	MSE	MAE	MSE	MAE
Electricity	96	**0.141**	**0.237**	0.158	0.258	0.144	0.240
	192	**0.157**	**0.252**	0.174	0.271	0.160	0.255
	336	**0.169**	**0.265**	0.182	0.280	0.176	0.273
	720	0.199	0.296	0.202	0.298	**0.198**	**0.294**
	Avg	**0.167**	**0.263**	0.179	0.277	0.170	0.267
PEMS07	12	**0.058**	**0.152**	0.061	0.160	0.062	0.161
	24	**0.072**	**0.172**	0.085	0.186	0.096	0.200
	48	**0.093**	**0.197**	0.121	0.226	0.170	0.239
	96	**0.112**	**0.214**	0.174	0.241	0.172	0.235
	Avg	**0.084**	**0.184**	0.110	0.203	0.125	0.209
TexasFreeze	12	**0.105**	**0.212**	0.137	0.286	0.146	0.284
	24	**0.179**	**0.296**	0.216	0.331	0.219	0.336
	48	**0.347**	0.431	0.389	**0.405**	0.393	0.407
	96	**0.628**	**0.619**	0.652	0.657	0.651	0.659
	Avg	**0.315**	**0.390**	0.349	0.420	0.352	0.422

Table S4. Ablation study on the proportion of variables processed by the Hankel-AUG module. Performance (MSE/MAE) is shown for multi-step forecasting on the TexasFreeze, Heatwave, and Antarctic Heat datasets. The best results for each metric and horizon are highlighted in bold.

Models		Hankelformer (FULL)		Hankel-AUG Ratio 0%		Hankel-AUG Ratio 25%		Hankel-AUG Ratio 50%		Hankel-AUG Ratio 75%	
Metric		MSE	MAE	MSE	MAE	MSE	MAE	MSE	MAE	MSE	MAE
TexasFreeze	12	**0.105**	**0.212**	0.224	0.327	0.143	0.256	0.158	0.235	0.133	0.221
	24	0.179	**0.296**	0.319	0.400	0.264	0.311	0.277	0.312	**0.178**	0.301
	48	**0.347**	**0.431**	0.455	0.498	0.376	0.464	0.382	0.459	0.352	0.444
	96	**0.628**	0.619	0.763	0.801	0.659	0.651	0.635	0.642	0.633	**0.614**
	Avg	**0.315**	**0.390**	0.440	0.507	0.361	0.421	0.363	0.435	0.324	0.395
Heatwave	12	**0.100**	**0.205**	0.199	0.261	0.182	0.244	0.147	0.231	0.125	0.219
	24	**0.170**	**0.276**	0.283	0.355	0.299	0.333	0.231	0.297	0.198	0.283
	48	**0.263**	0.358	0.306	0.404	0.332	0.394	0.278	**0.355**	0.271	0.357
	96	**0.451**	**0.466**	0.519	0.505	0.505	0.486	0.483	0.472	0.462	0.469
	Avg	**0.246**	**0.326**	0.327	0.381	0.330	0.364	0.285	0.339	0.264	0.332
Antarctic Heat	12	**0.147**	**0.263**	0.214	0.305	0.209	0.305	0.198	0.291	0.162	**0.263**
	24	**0.203**	**0.320**	0.297	0.384	0.296	0.376	0.241	0.352	0.244	0.349
	48	**0.272**	**0.382**	0.346	0.443	0.341	0.451	0.324	0.411	0.302	0.384
	96	**0.316**	**0.419**	0.408	0.462	0.415	0.469	0.367	0.440	0.365	0.432
	Avg	**0.235**	**0.346**	0.316	0.399	0.315	0.400	0.283	0.364	0.268	0.357

Table S5. Training efficiency comparison. Experiments are conducted on a generated multivariate time series dataset with seven variables; both input and prediction lengths are set to 96. Measurements were taken on an NVIDIA RTX 3080Ti GPU with a fixed batch size.

Models	Parameters (M)	Inference Time (ms)	Training Iteration (ms)	Peak GPU Memory (MB)	FLOPs (G)
Hankelformer	1.25	7.11	21.39	38.47	0.22
iTransformer	1.21	3.15	10.07	38.27	0.11
PatchTST	1.34	3.66	10.31	51.45	0.80
WPMixer	0.74	5.43	15.44	90.32	0.14
TimeMixer	1.13	10.99	35.36	149.63	11.10
TiDE	0.41	18.14	55.21	45.77	0.24
TimesNet	299.94	263.49	1081.43	1412.33	2328.39
CoST	102.34	889.24	784.40	508.01	78.26
SimMTM	2.37	8.59	18.32	138.09	6.42

Table S6. The performance of Hankelformer on the New York Precipitation dataset. All predictions are conducted based on past 96 time points. The best performance is highlighted in bold.

Models	MSE	SEDI
Hankelformer	**1.357**	**0.570**
iTransformer	1.365	0.545
PatchTST	1.369	0.526
WPMixer	1.371	0.537
TimeMixer	1.370	0.552
TiDE	1.382	0.502
TimesNet	1.372	0.511

Table S7. Sensitivity analysis on the contrastive weight λ. Performance (MSE/MAE) for multi-step temperature prediction on the Electricity, PEMS07 and TexasFreeze datasets. Best results are in bold.

Contrastive Weight λ	Electricity		PEMS07		TexasFreeze	
	MSE	MAE	MSE	MAE	MSE	MAE
0.00005	0.147	0.247	0.169	0.267	0.068	0.175
0.0001	0.15	0.252	0.171	0.268	0.071	0.179
0.001	**0.141**	**0.237**	0.164	0.265	0.069	0.175
0.005	0.142	**0.237**	0.169	0.266	0.068	0.175
0.01	0.143	0.239	0.175	0.275	**0.067**	0.173
0.03	**0.141**	0.24	**0.139**	0.245	0.07	0.178
0.05	0.155	0.255	0.141	**0.239**	0.071	0.178
0.1	0.15	0.253	0.166	0.267	0.071	**0.169**
0.375	0.148	0.244	0.201	0.306	0.073	0.183

Table S8. Sensitivity analysis on the Hankel window size W. Performance (MSE/MAE) for multi-step temperature prediction on the Electricity, PEMS07 and TexasFreeze datasets. Best results are in bold.

Hankel window size W	Electricity		PEMS07		TexasFreeze	
	MSE	MAE	MSE	MAE	MSE	MAE
1	0.167	0.267	0.166	0.264	0.168	0.371
3	0.163	0.267	0.304	0.39	0.163	0.324
5	0.165	0.27	0.253	0.352	0.144	0.267
10	0.166	0.265	0.234	0.335	0.14	0.239
12	0.155	0.257	0.267	0.363	0.126	0.221
15	0.154	0.257	0.266	0.346	**0.105**	**0.212**
18	**0.141**	**0.237**	0.176	0.271	0.111	0.214
24	0.143	0.239	0.183	0.249	0.127	0.223
32	0.144	0.240	**0.112**	**0.214**	0.144	0.233

Table S9. Sensitivity analysis on the contrastive temperature τ. Performance (MSE/MAE) for multi-step temperature prediction on the Electricity, PEMS07 and TexasFreeze datasets. Best results are in bold.

Temperature τ	Electricity		PEMS07		Texas Freeze	
	MSE	MAE	MSE	MAE	MSE	MAE
0.1	0.142	0.242	0.118	0.216	0.107	0.223
0.2	0.157	0.261	**0.110**	0.215	0.109	0.221
0.3	0.151	0.252	0.111	0.213	0.111	0.214
0.4	0.142	0.240	0.116	**0.209**	0.108	0.220
0.5	**0.141**	**0.237**	0.112	0.214	0.105	0.212
0.6	0.142	0.238	0.120	0.221	0.106	**0.209**
0.7	**0.141**	**0.237**	0.122	0.218	0.112	0.213
0.8	0.142	0.239	0.119	0.216	**0.104**	0.210
0.9	0.143	0.239	0.122	0.220	0.105	0.211
1.0	**0.141**	**0.237**	0.118	0.216	0.113	0.219

Table S10. Performance comparison of W obtained using FNN and grid search. Best results are in bold.

Hankel	Best W		FNN W	
Window size W	MSE	MAE	MSE	MAE
Electricity	**0.141**	**0.237**	0.165	0.270
PEMS07	**0.112**	**0.214**	0.196	0.300
TexasFreeze	**0.105**	**0.212**	0.109	0.216

Table S11. Complete parameter settings of public dataset experiments.

Dataset		Encoder layer	Encoder dimension	FFN dimension	Hankel window size	Contra. Loss weight	Learning rate	Batch size
Electricity	96	3	512	512	18	0.03	0.0005	16
	192	3	512	512	24	0.01	0.0005	16
	336	3	512	512	32	0.1	0.0005	16
	720	3	512	512	32	0.1	0.0005	16
Traffic	96	4	512	512	3	0.1	0.001	16
	192	4	512	512	12	0.07	0.001	16
	336	4	512	512	12	0.07	0.001	16
	720	4	512	512	12	0.07	0.001	16
PEMS03	12	4	512	512	1	0.03	0. 001	64
	24	4	512	512	1	0.03	0. 001	64
	48	4	512	512	1	0.03	0. 001	64
	96	4	512	512	1	0.03	0. 001	64
PEMS04	12	4	1024	1024	1	0.05	0.0005	32
	24	4	1024	1024	1	0.05	0.0005	32
	48	4	1024	1024	1	0.05	0.0005	32
	96	4	1024	1024	1	0.05	0.0005	32
PEMS07	12	2	512	512	1	0.03	0.001	32
	24	2	512	512	1	0.03	0.001	32
	48	2	512	512	1	0.03	0.001	32
	96	2	512	512	1	0.03	0.001	32
PEMS08	12	4	512	512	4	0.05	0.001	64
	24	4	512	512	4	0.05	0.001	64
	48	4	512	512	4	0.05	0.001	64
	96	4	512	512	4	0.05	0.001	64

Table S12. Complete parameter settings of 90D-Lorenz system dataset experiments. The prediction window is fixed at 96 time steps.

Noise Level	Encoder layer	Encoder dimension	FFN dimension	Hankel window size	Contra. Loss weight	Learning rate	Batch size
0	4	512	512	3	0.375	0.0014	128
0.1	4	512	512	3	0.375	0.0014	128
0.2	4	512	512	3	0.375	0.0014	128
0.3	4	512	512	3	0.375	0.0014	128
0.4	4	512	512	3	0.375	0.0014	128
0.5	4	512	512	3	0.375	0.0014	128
0.6	4	512	512	3	0.375	0.0014	128
0.7	4	512	512	3	0.375	0.0014	128
0.8	4	512	512	3	0.375	0.0014	128
0.9	4	512	512	3	0.375	0.0014	128
1.0	4	512	512	3	0.375	0.0014	128

Table S13. Complete parameter settings of extreme weather prediction experiments.

Dataset		Encoder layer	Encoder dimension	FFN dimension	Hankel window size	Contra. Loss weight	Learning rate	Batch size
Freeze	12	2	512	512	15	0.005	0.0005	64
	24	2	512	512	5	0.005	0.0005	64
	48	2	512	512	10	0.01	0.0003	64
	96	2	512	512	20	0.001	0.0003	64
Heatwave	12	2	512	512	2	0.00005	0.0012	64
	24	2	512	512	10	0.01	0.0001	64
	48	2	512	512	21	0.01	0.0005	64
	96	2	512	512	10	0.01	0.0001	64
Antarctic Heat	12	2	512	512	3	0.0001	0.00015	64
	24	2	512	512	3	0.0001	0.00015	64
	48	2	512	512	3	0.00005	0.0001	64
	96	2	512	512	3	0.0001	0.00015	64
